# Material Basis and Mechanisms of Action of PuRenDan in the Treatment of Type 2 Diabetes Mellitus: An Integrated Network Pharmacology and Molecular Simulation Study

**DOI:** 10.3390/ph19071107

**Published:** 2026-07-17

**Authors:** Wenshuai Yang, Gaojie Ouyang, Wenwen Zhou, Binan Lu, Zongran Pang

**Affiliations:** 1 Key Laboratory of Ethnic Medicine in Ministry of Education, School of Pharmacy, Minzu University of China, Beijing 100081, China; 25302181@muc.edu.cn (W.Y.);; 2 College of Acupuncture and Orthopedics, Hubei University of Chinese Medicine, Wuhan 430065, China; tow_0810@163.com

**Keywords:** PuRenDan, type 2 diabetes mellitus, network pharmacology, molecular dynamics simulation, β-sitosterol, MM/PBSA

## Abstract

**Background/Objectives**: Type 2 diabetes mellitus (T2DM) is a chronic multifactorial metabolic disorder requiring multi-target therapeutic strategies. This study aimed to predict the potential material basis, key targets and molecular mechanisms by which PuRenDan (PRD) may act against T2DM through an integrated network pharmacology and molecular simulation approach. **Methods**: Active compounds of PRD were screened from TCMSP, HERB 2.0 and the literature, and compound-related targets were predicted using TCMSP, SwissTargetPrediction and PharmMapper. T2DM-associated targets were collected from OMIM, DrugBank, DisGeNET, HPO, ClinPGx and GeneCards to obtain drug–disease intersection targets. Cytoscape was used to construct herb–compound–target and protein–protein interaction (PPI) networks, followed by Gene Ontology (GO) and Kyoto Encyclopedia of Genes and Genomes (KEGG) enrichment analyses. Molecular docking was performed using AutoDock Vina1.1.2, and representative ligand–receptor complexes were further assessed by 100 ns molecular dynamics (MD) simulations and molecular mechanics/Poisson–Boltzmann surface area (MM/PBSA) binding free-energy analysis. **Results**: A total of 163 active compounds, 597 PRD-related targets, 9138 T2DM-associated targets and 483 intersection targets were identified. β-sitosterol, emodin, quercetin, kaempferol and formononetin were predicted as major active compounds, whereas AKT1, TP53, SRC, IL6, TNF, EGFR and ESR1 were identified as disease-related network hubs. KEGG enrichment highlighted the PI3K-Akt, MAPK, HIF-1, FoxO, mTOR, AGE-RAGE and TNF signalling pathways. Docking predicted a comparatively favourable multi-target binding tendency for β-sitosterol. MD and MM/PBSA analyses further suggested favourable dynamic stability for β-sitosterol-TNF, β-sitosterol-AKT1, β-sitosterol-SRC and emodin-EGFR complexes, with β-sitosterol-TNF showing the lowest predicted binding free energy among the simulated systems. **Conclusions**: These in silico findings suggest that PRD may regulate T2DM-related inflammatory, insulin-signalling, oxidative-stress and metabolic networks through coordinated multi-compound, multi-target and multi-pathway actions. β-sitosterol may represent an important candidate material basis of PRD, with TNF, AKT1, SRC and EGFR as potential key targets. These conclusions remain predictive and require validation in biochemical, cellular and animal experiments.

## 1. Introduction

Type 2 diabetes mellitus (T2DM) is a chronic metabolic disease characterised by insulin resistance and relative insulin secretory insufficiency. According to the most recent data published in The Lancet in November 2024, approximately 828 million adults worldwide had diabetes in 2022, of whom 85–95% were affected by T2DM [1]. The harm caused by T2DM is not limited to dysregulated glycaemia; chronic inflammation and oxidative stress also accelerate multi-organ injury. Studies have shown that the risk of cardiovascular events is markedly higher in patients with T2DM than in non-diabetic populations, and long-term mortality is approximately 50% higher among patients with T2DM than among those without T2DM [2]. The cumulative incidence of microvascular complications, including retinopathy and nephropathy, also increases with disease duration [3,4]. T2DM has therefore become a major global public health challenge.

PuRenDan (PRD) is a clinical traditional Chinese medicine formula used for the treatment of T2DM and comprises Ginseng Radix et Rhizoma, Momordicae Charantiae Fructus, Salviae Miltiorrhizae Radix et Rhizoma, Puerariae Lobatae Radix and Polygoni Multiflori Radix Praeparata. Previous clinical practice and experimental studies by our research group indicated that PRD has favourable effects on improving glucose and lipid metabolic disorders, alleviating insulin resistance, restoring abnormal insulin signalling, repairing pancreatic β-cell injury and increasing β-cell mass, suggesting potential in the prevention and treatment of T2DM and its complications. Earlier cellular and animal studies demonstrated experimentally that PRD may improve T2DM-related pathological processes by regulating the PI3K-Akt, MAPK, FoxO, mTOR and TNF signalling pathways and by affecting the expression levels of FOXO, TNF and MTOR proteins, thereby providing pharmacodynamic support for its effects in T2DM [5,6,7,8]. However, although these previous studies established the pharmacological effects and pathway-level involvement of PRD, the specific compounds responsible for these effects, their relative target contribution and their dynamic ligand–target binding characteristics remained unclear. On the basis of this prior experimental evidence, the present study further integrated network pharmacology, molecular docking, molecular dynamics simulation and MM/PBSA binding free-energy analysis to investigate PRD against T2DM from the perspective of compound–target–pathway–molecular interaction relationships. In particular, this study aimed to computationally identify candidate key contributors and to assess the binding stability of representative compound–target complexes. The results are intended to prioritise candidate compounds and targets for subsequent experimental validation rather than to replace direct pharmacological verification.

## 2. Results

### 2.1. Screening of Active Compounds and Targets of PuRenDan

By integrating searches of TCMSP and HERB 2.0 with literature retrieval, 20 active compounds were identified from Ginseng Radix et Rhizoma (RS1-RS20), 71 from Momordicae Charantiae Fructus (KG1-KG71), 55 from Salviae Miltiorrhizae Radix et Rhizoma (DS1-DS55), 4 from Puerariae Lobatae Radix (GG1-GG4) and 13 from Polygoni Multiflori Radix Praeparata (ZSW1-ZSW13). Four shared active compounds that were identified and coded as A1, B1, C1 and D1. These shared compounds are shown in Table 1. After removal of duplicate targets identified from TCMSP, SwissTargetPrediction and the literature, 597 PRD-related targets were obtained.

### 2.2. Acquisition of T2DM-Associated Disease Targets

T2DM-associated targets were collected from OMIM, DrugBank, GeneCards and other databases, together with literature retrieval. To reduce reliance on a single database, targets appearing in at least two databases were retained, and GeneCards targets with a relevance score above the median were used as supplementary T2DM-associated disease targets. In total, 9138 T2DM-associated targets were screened. This strategy was intended to balance disease-target coverage and downstream network construction; however, because broad disease databases may still include weakly associated or pleiotropic genes, subsequent PPI topology, pathway enrichment, molecular simulation and external pharmacological plausibility checks were used to further focus the candidate target set.

### 2.3. Identification of Compound–Disease Intersection Targets and Network Construction

**Figure 1:**
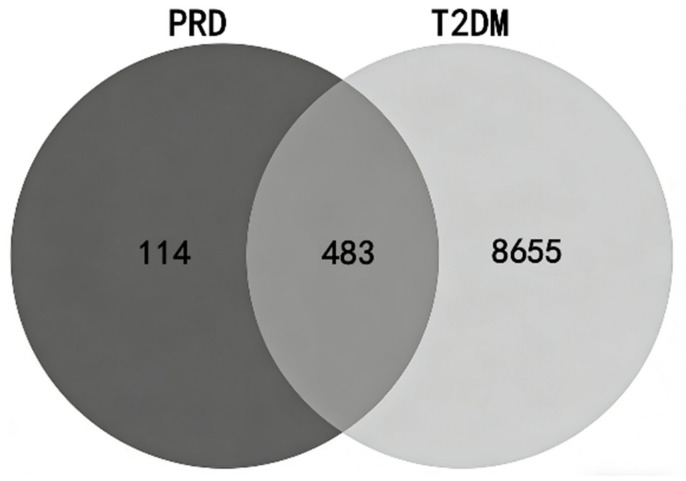
Venn diagram showing the intersection between PuRenDan-related targets and T2DM-associated disease targets.

**Figure 2:**
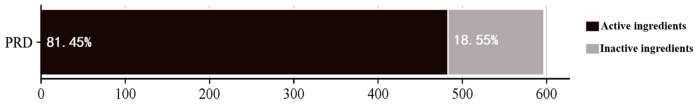
Proportion of T2DM-related intersection targets among PuRenDan-related targets.

The PRD-related targets and T2DM-associated disease targets were intersected using a Venn diagram (Figure 1), yielding 483 putative therapeutic targets of PRD against T2DM, The overlapping colored regions in the Venn diagram represent the intersecting targets shared by PRD and T2DM. The overlap between PRD-related targets and T2DM-associated targets was relatively high (Figure 2), suggesting that PRD active compounds may cover multiple pathological processes related to T2DM. However, because the disease targets were collected from broad data sources, PPI topological analysis, pathway enrichment and molecular simulation were required to further focus on key targets.

As an external pharmacological plausibility check, established therapeutic targets of marketed antidiabetic drugs were compared with the 483 intersection targets. The DPP-4 inhibitor target DPP4, SGLT2 inhibitor target SLC5A2, thiazolidinedione target PPARG, α-glucosidase inhibitor-related target MGAM and insulin-related target INSR were all present in the intersection set. In addition, several enriched PRD pathways overlapped with known antidiabetic drug-related signalling networks, including INSR-mediated PI3K-Akt, MAPK, FoxO and mTOR signalling, incretin-related PI3K-Akt/FoxO/mTOR regulation, and SGLT2 inhibitor-associated inflammatory, oxidative-stress, AGE-RAGE and mTOR/AMPK-related processes [9,10,11,12,13,14,15,16,17,18,19,20,21,22]. This comparison supports the pharmacological plausibility of the predicted intersection targets, but it should be interpreted as evidence of mechanistic convergence rather than proof that PRD acts through the same mechanisms as these drugs.

**Figure 3:**
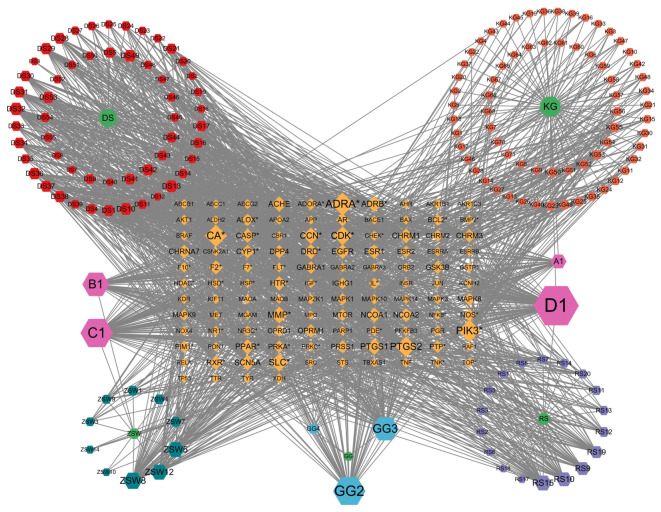
Herb–compound–active target network.

An herb–compound–active target network was constructed using Cytoscape 3.10.4 (Figure 3). For clarity, only active targets with degree values greater than 5 are displayed. To ensure sufficient clarity of the network diagram, several target series were simplified and marked with an asterisk (*). For instance, CA* stands for CA1 to CA9. Detailed data on the corresponding targets of each component are provided in Supplementary Table S1. Screened Components and Targets of Purendan. In the network, hexagons of different colours represent active compounds from different component medicines, green hexagons represent the component medicines of PRD and orange diamonds represent active targets. Larger fonts and nodes, together with denser connecting lines, indicate higher degree values and therefore a more central role of the compound or target in the therapeutic network of PRD against T2DM. Degree, betweenness and closeness values of the active compounds were calculated using the Analyze Network function and imported into SPSS 27 for scoring and ranking. The seven compounds with the highest scores were D1, C1, GG2, B1, GG3, ZSW8 and A1 (Table 2). These compounds were associated with more active targets and may therefore be more important in the multi-target synergistic treatment of T2DM; they were preliminarily screened as core compounds.

### 2.4. PPI Network Analysis and Identification of Core Target Clusters

**Figure 4:**
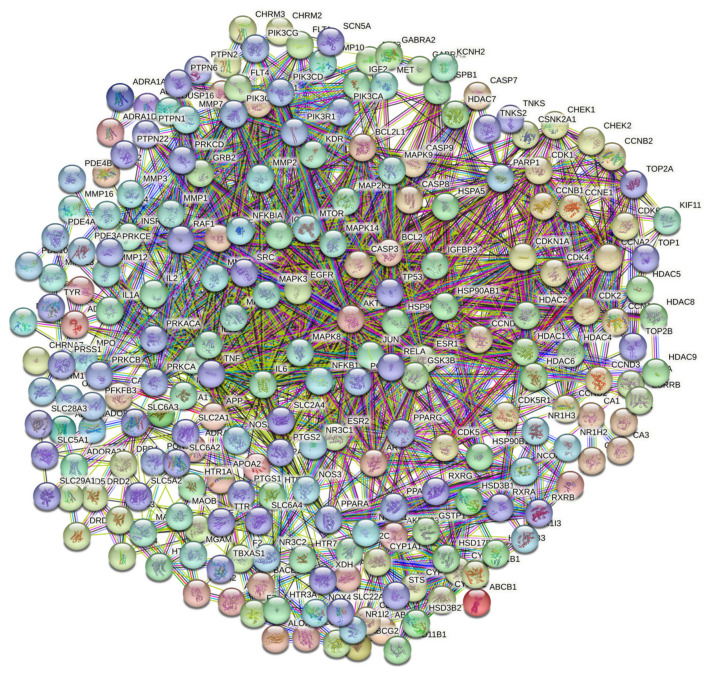
PPI network of active targets of PuRenDan.

PPI analysis of the 483 active targets was performed using the STRING database, and the resulting PPI network is shown in Figure 4. Targets closer to the centre of the network and linked by denser edges were considered to have more direct interactions with other proteins.

The TSV file of the PPI network was imported into Cytoscape 3.10.4 for degree, betweenness and closeness analyses. The results were then imported into SPSS 27 for scoring. According to the ranking, the top 15 targets were preliminarily defined as core targets: AKT1, TP53, SRC, IL6, TNF, EGFR, ESR1, CTNNB1, IL1B, MYC, CASP3, PTGS2, STAT3, HSP90AA1 and PPARG, with degree values of 267, 258, 210, 251, 251, 224, 208, 212, 215, 213, 218, 182, 214, 192 and 180, respectively (Table 3).

**Figure 5:**
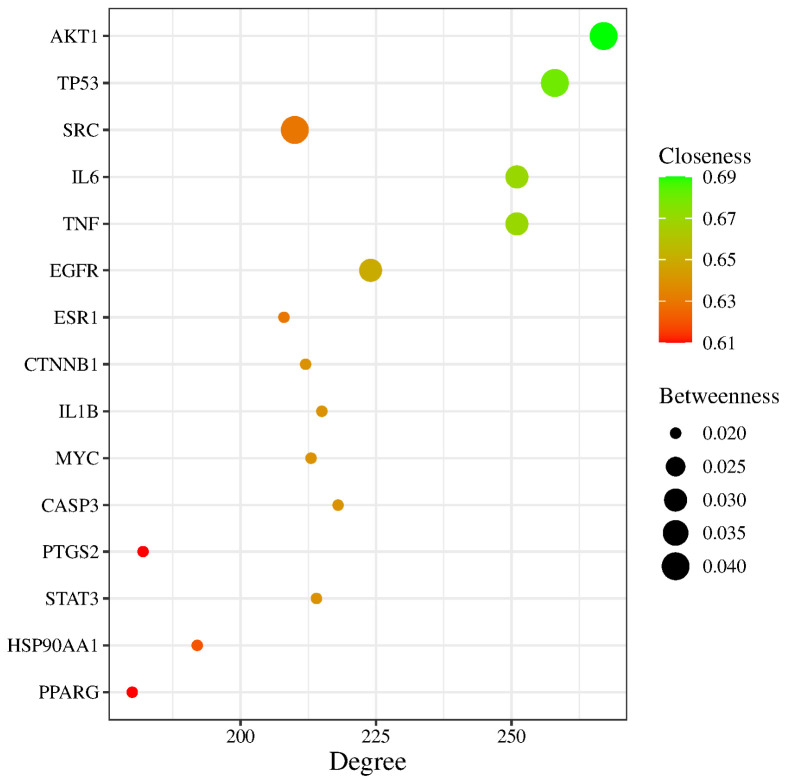
Bubble plot of core targets of PuRenDan.

In the bubble plot (Figure 5), the x-axis represents degree. Node colour follows a red-to-green gradient corresponding to increasing closeness values, with the darkest green node indicating the highest closeness value. Node size is positively associated with betweenness.

### 2.5. GO and KEGG Enrichment Analyses

**Figure 6:**
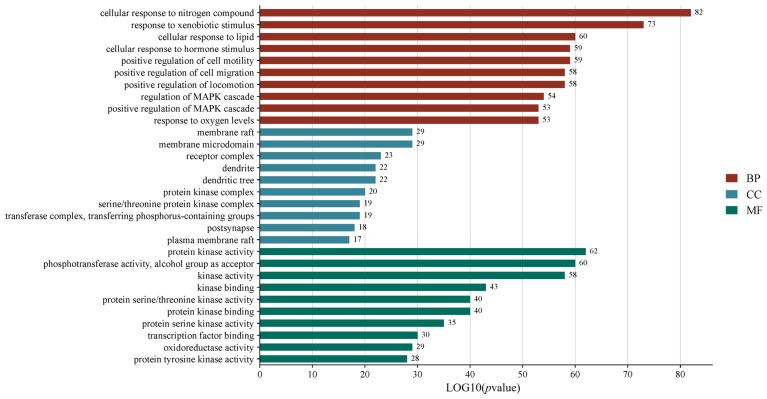
GO-BP, GO-CC and GO-MF enrichment analysis of active targets of PuRenDan.

GO enrichment analysis, including biological process (BP), cellular component (CC) and molecular function (MF), and KEGG enrichment analysis were performed for the 483 active targets using Metascape. GO-BP terms were mainly enriched in responses to nitrogen compounds, xenobiotic stimuli, lipids and hormone stimuli, positive regulation of cell motility and migration, positive regulation of the MAPK cascade and response to oxygen levels. GO-CC terms were mainly enriched in membrane structures, including membrane rafts, plasma membrane rafts and membrane microdomains, as well as receptor complexes, protein kinase complexes, serine/threonine protein kinase complexes, phosphorus-containing group transferase complexes, postsynaptic membranes and dendritic structures. GO-MF terms were mainly enriched in kinase-related activities, including kinase activity, protein kinase activity, protein serine kinase activity, protein serine/threonine kinase activity, protein tyrosine kinase activity and phosphotransferase activity with an alcohol group as acceptor, as well as binding activities, including kinase binding, protein kinase binding and transcription factor binding, and oxidoreductase activity (Figure 6). Detailed information on the top 10 terms from GO enrichment analysis is provided in the Supplementary Materials: the top 10 screened GO-BP, GO-CC and GO-MF pathways are presented in Table S2, Table S3 and Table S4, respectively.

**Figure 7:**
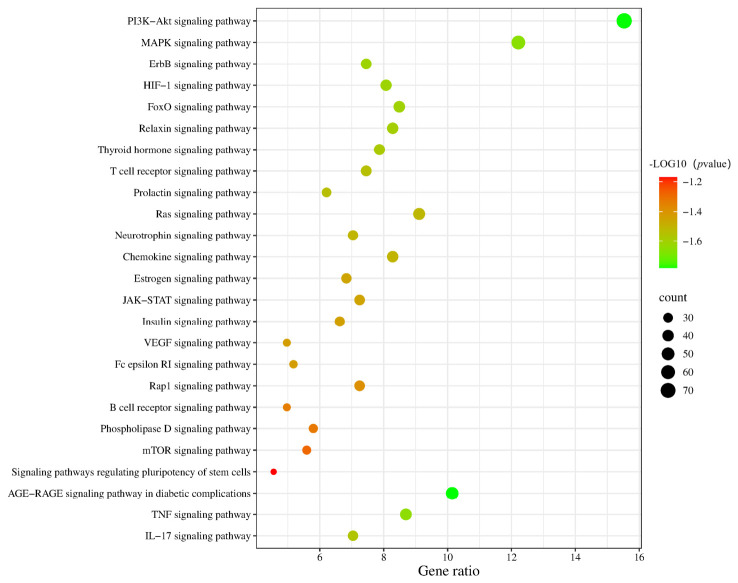
KEGG pathway enrichment analysis of active targets of PuRenDan.

**Figure 8:**
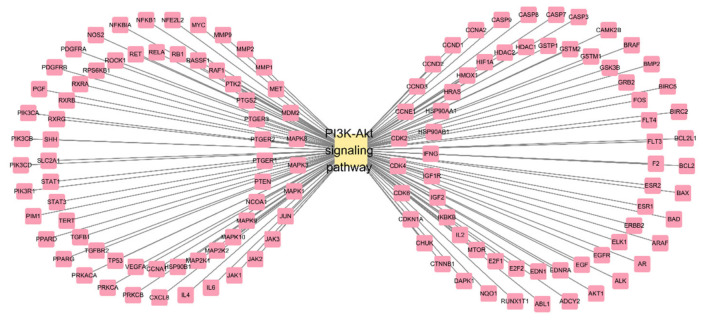
Target-PI3K-Akt signalling pathway network.

KEGG enrichment analysis indicated that PRD may treat T2DM mainly through regulation of the PI3K-Akt signalling pathway, MAPK signalling pathway, HIF-1 signalling pathway, FoxO signalling pathway, relaxin signalling pathway, thyroid hormone signalling pathway, oestrogen signalling pathway, chemokine signalling pathway, mTOR signalling pathway, AGE-RAGE signalling pathway in diabetic complications and TNF signalling pathway (Figure 7). Table S5. The information of the top 25 KEGG pathways obtained through screening. Node colour follows a red-to-green gradient corresponding to increasing −log10(*p*) values; darker green indicates higher enrichment significance. Node size represents the number of active targets involved in each pathway, with larger nodes indicating more targets. Among all pathways, the PI3K-Akt signalling pathway contained the largest number of active targets, suggesting that it may play a particularly important role in the treatment of T2DM by PRD. To visualise the molecular mechanism by which PRD regulates the PI3K-Akt signalling pathway in T2DM, a target-PI3K-Akt signalling pathway network was further constructed (Figure 8). Pink nodes represent PRD-related targets acting on the PI3K-Akt pathway, including AKT1, AR, FOS, MTOR, IL6 and NFKB1. The top 25 KEGG pathways and their corresponding targets were imported into Cytoscape 3.10.4 to construct a compound–target–pathway network, followed by degree, betweenness and closeness analyses. After scoring in SPSS 27, the top seven compounds in the compound–target–pathway network were quercetin, kaempferol, inermin, emodin, luteolin, physcion and formononetin (Table 4).

### 2.6. Final Screening of Core Compounds

The scores of the preliminarily identified core compounds and the KEGG-derived core compounds were imported into SPSS 27 for rescoring. Specifically, the relevant topological indicators were standardised as Z-scores using the SPSS option “save standardised scores as variables”, and the comprehensive score was calculated as Score = ZBetweenness + ZCloseness + ZDegree. This standardised additive score enabled indicators with different scales and ranges to be combined and avoided dominance by the raw Degree value. A higher score indicates that the node is more central across the selected topological dimensions. Negative final scores indicate that the corresponding node was below the mean of the analysed node set after standardisation; they do not indicate negative biological activity or an inhibitory pharmacological effect. According to the final ranking, the top seven compounds were defined as final core compounds: quercetin, kaempferol, formononetin, β-sitosterol, emodin, inermin and luteolin (Table 5).

### 2.7. Molecular Docking Results

**Figure 9:**
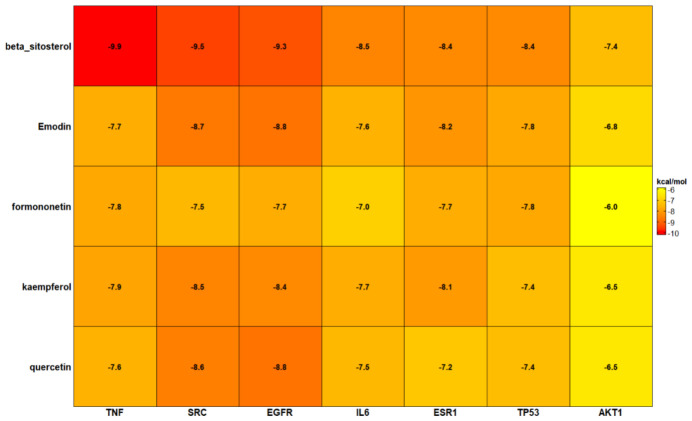
Heatmap of molecular docking binding energies.

**Figure 10:**
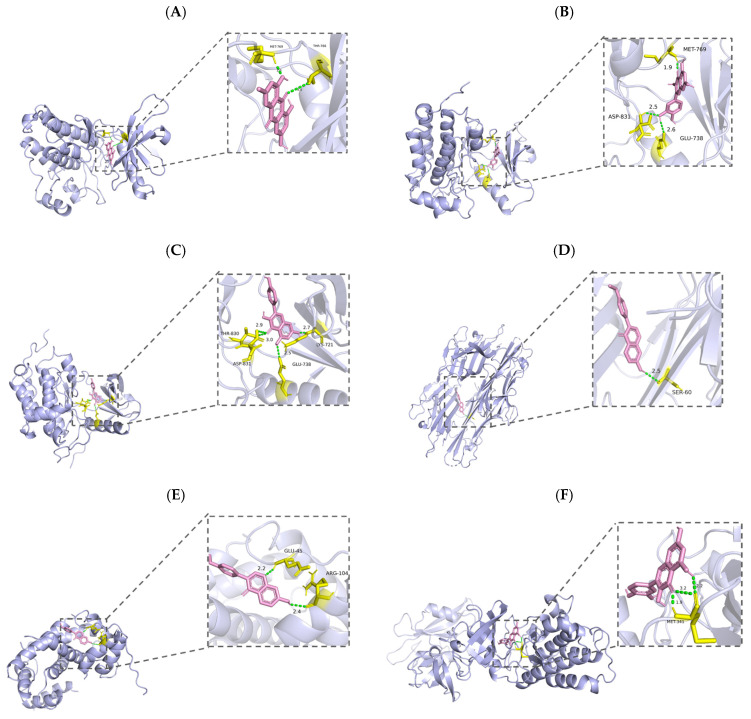

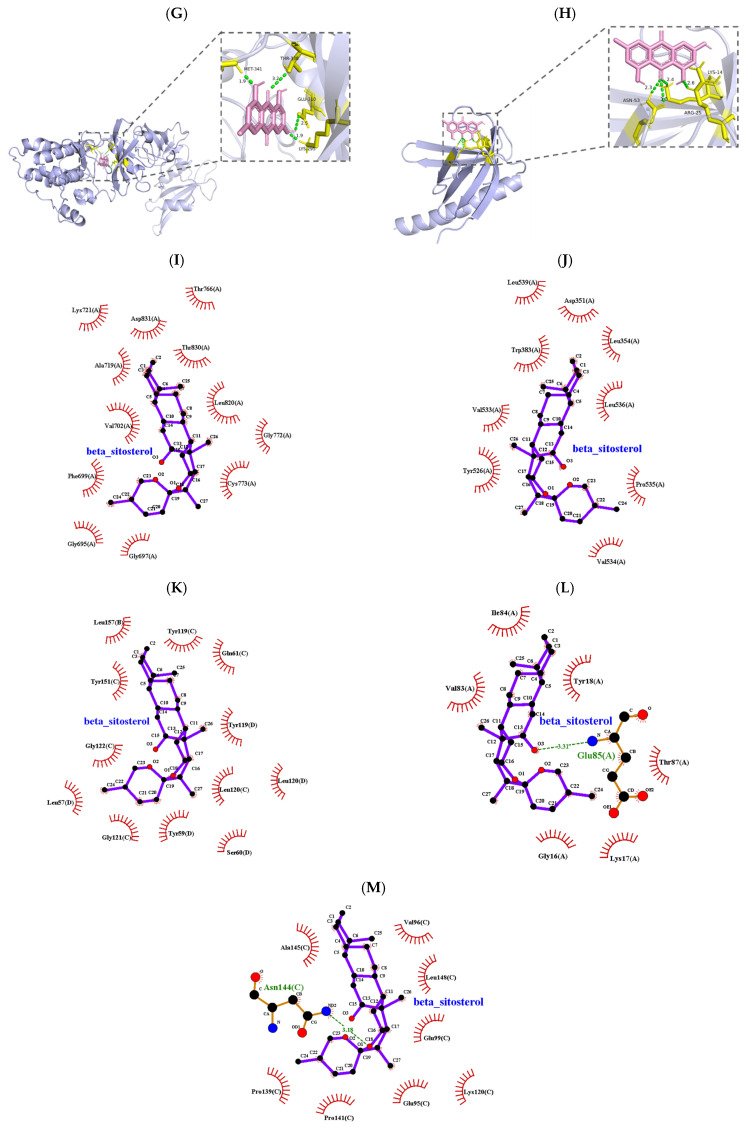
Molecular docking visualisation results. Panels (**A**–**H**) show three-dimensional ligand–receptor binding conformations generated by PyMOL3.1.8, mainly displaying hydrogen bonds and binding-pocket positions; panels (**I**–**M**) show two-dimensional LigPlus2.3.1 interaction diagrams of β-sitosterol complexes, mainly displaying hydrophobic residue encapsulation and non-hydrogen-bond interactions. (**A**) Emodin-EGFR (1M17); (**B**) quercetin-EGFR (1M17); (**C**) kaempferol-EGFR (1M17); (**D**) formononetin-TNF (2AZ5); (**E**) formononetin-TP53 (1DT7); (**F**) quercetin-SRC (1Y57); (**G**) kaempferol-SRC (1Y57); (**H**) emodin-AKT1 (2UZR); (**I**) β-sitosterol-EGFR (1M17); (**J**) β-sitosterol-ESR1 (1SJ0); (**K**) β-sitosterol-TNF (2AZ5); (**L**) β-sitosterol-AKT1 (2UZR); (**M**) β-sitosterol-IL6 (4J4L).

Pairwise molecular docking was performed between the key compounds and key targets, and the corresponding binding energies are shown in Table 6 and visualised as a heatmap in Figure 9. The heatmap indicated that β-sitosterol showed the best overall binding with the seven key targets. The optimal docking conformations were visualised using PyMOL3.1.8 and LigPlus2.3.1; for receptors whose optimal docking ligand was β-sitosterol, the second-best docking conformation was also visualised (Figure 10).

A docking binding energy below −5.0 kcal/mol is generally considered to indicate potential ligand–receptor binding activity, whereas a binding energy below −7.0 kcal/mol suggests a favourable predicted binding tendency. However, docking scores are computational ranking indices rather than experimental affinity constants and should not be interpreted alone as proof of biological efficacy or target inhibition. In this study, the binding energies of the five key compounds with the seven core targets ranged from −6.0 to −9.9 kcal/mol. β-sitosterol showed comparatively favourable docking energies with all seven key targets: AKT1 (2UZR), ESR1 (1SJ0), TP53 (1DT7), IL6 (4J4L), EGFR (1M17), SRC (1Y57) and TNF (2AZ5), with values of −7.4, −8.4, −8.4, −8.5, −9.3, −9.5 and −9.9 kcal/mol, respectively. These results suggest good spatial complementarity and a potential multi-target recognition tendency. In particular, the β-sitosterol-EGFR result should be interpreted as a moderate predicted interaction within the PRD multi-target network, not as evidence that β-sitosterol behaves as a potent or selective EGFR tyrosine kinase inhibitor comparable to clinical EGFR-TKIs.

Docking visualisation showed that the five core compounds formed hydrogen bonds and hydrophobic interactions with specific amino acid residues of the seven core target proteins. Hydrogen-bond distances ranged from 1.9 to 3.5 Å, indicating close ligand–receptor hydrogen-bonding interactions. Of note, β-sitosterol, which showed the strongest binding among the ligands, did not display obvious hydrogen bonds in PyMOL3.1.8. However, LigPlus2.3.1 analysis showed that β-sitosterol was surrounded and embedded by numerous hydrophobic amino acid residues, forming a stable hydrophobic interaction network. In this study, two-dimensional LigPlus2.3.1 interaction diagrams were therefore used to present β-sitosterol complexes, rather than only applying the same three-dimensional PyMOL3.1.8 hydrogen-bond visualisation used for polyphenolic compounds. This strategy was chosen because β-sitosterol has a typical hydrophobic steroidal skeleton with few polar groups capable of forming stable hydrogen bonds. Its binding to TNF, AKT1, SRC, EGFR, ESR1 and IL6 was mainly characterised by hydrophobic residue encapsulation, van der Waals interactions and aromatic/aliphatic residue contacts rather than persistent hydrogen-bond dominance. Sole reliance on PyMOL3.1.8 hydrogen-bond display and three-dimensional binding conformations could underestimate non-polar interactions between β-sitosterol and receptor hydrophobic pockets and would not clearly show the surrounding hydrophobic residue network. Therefore, after three-dimensional conformational inspection, LigPlus2.3.1 was further used to visualise β-sitosterol complexes in two dimensions to highlight hydrophobic burial and non-hydrogen-bond interactions. This visualisation approach is consistent with the molecular structure of β-sitosterol and the MM/PBSA results, indicating that its stronger binding stability mainly arises from MM, SA and hydrophobic/van der Waals contributions rather than from a numerical advantage in hydrogen bonds.

### 2.8. Molecular Dynamics Simulation Results

#### 2.8.1. MD Trajectory Analysis

Eight representative complexes were selected for MD simulation before production simulations were performed based on integrated consideration of docking affinity, network centrality, compound representativeness and system parameterisation feasibility: TNF-β-sitosterol, AKT1-β-sitosterol, ESR1-β-sitosterol, EGFR-β-sitosterol, SRC-β-sitosterol, EGFR-emodin, EGFR-kaempferol and AKT1-quercetin. These complexes were selected to support two comparative purposes: comparing the same candidate compound, β-sitosterol, across different core targets, and comparing different representative PRD compounds acting on the same targets, especially EGFR and AKT1. The selection was not made by excluding complexes according to post hoc MD outcomes such as RMSD or MM/PBSA values. Time-dependent changes in SASA, Rg, RMSD, RMSF and hydrogen-bond number were visualised according to comparisons between the same ligand with different receptors and between the same receptor with different ligands.

**Figure 11:**
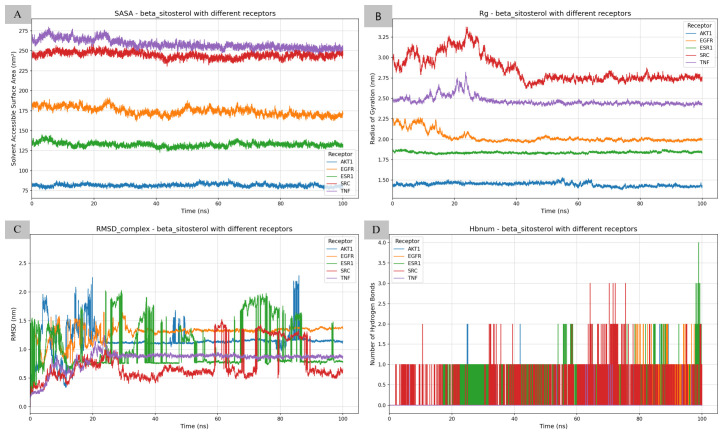

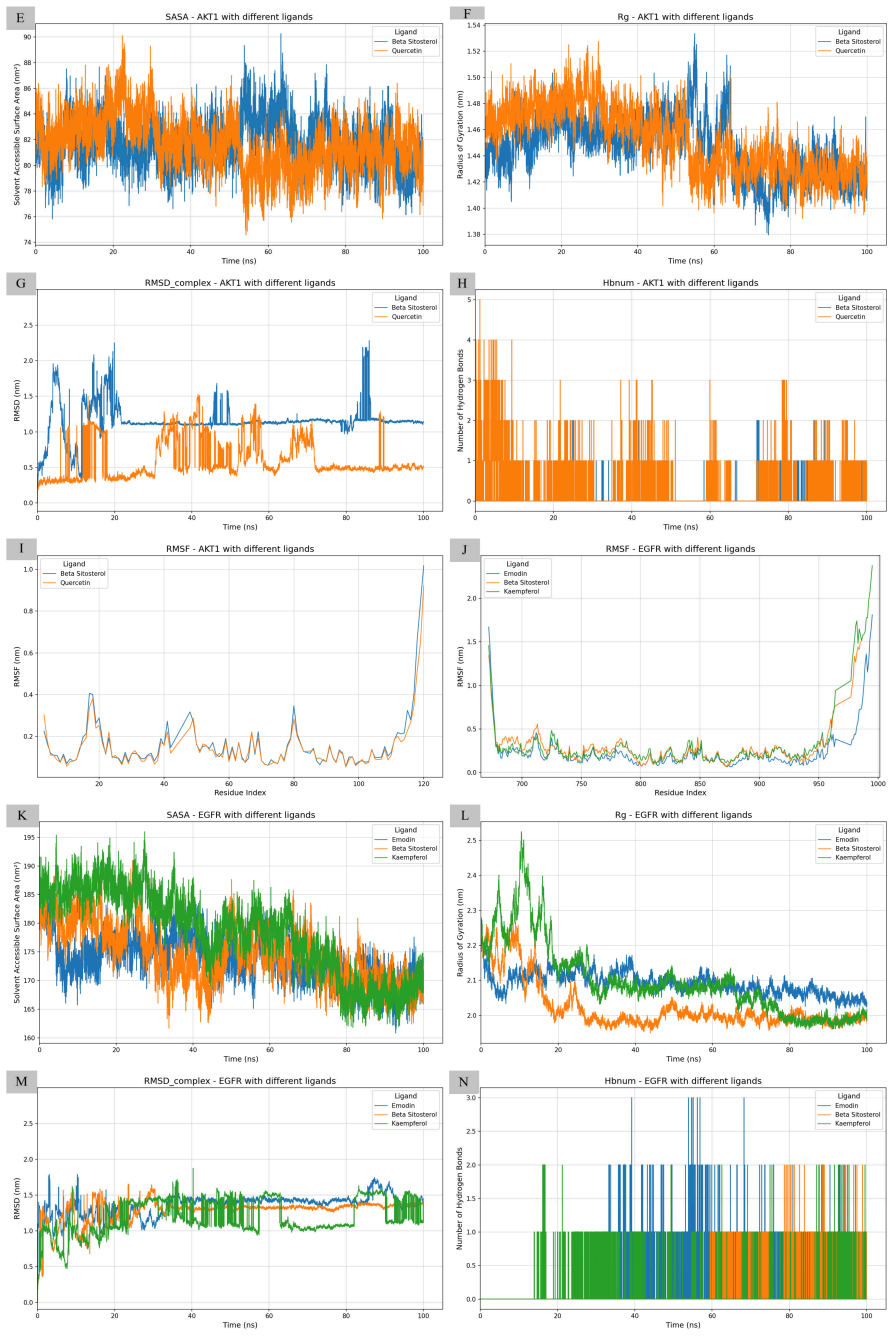
Molecular dynamics trajectory analyses of representative ligand–receptor complexes. (**A**–**D**) SASA, Rg, RMSD and hydrogen-bond number of β-sitosterol complexes with AKT1, EGFR, ESR1, SRC and TNF; (**E**–**I**) SASA, Rg, RMSD, hydrogen-bond number and RMSF of β-sitosterol-AKT1 and quercetin-AKT1; (**J**–**N**) RMSF, SASA, Rg, RMSD and hydrogen-bond number of emodin-EGFR, β-sitosterol-EGFR and kaempferol-EGFR.

Figure 11A–D compares the changes in SASA, Rg, hydrogen-bond number and RMSD for β-sitosterol with AKT1, EGFR, ESR1, SRC and TNF.

SASA analysis showed that β-sitosterol-AKT1 had the lowest SASA, approximately 80 nm^2^, consistent with its smallest Rg and compact structure. The SASA of β-sitosterol-EGFR decreased from approximately 180 nm^2^ to 168–172 nm^2^, suggesting reduced solvent exposure that may be accompanied by ligand burial or pocket contraction. β-sitosterol-ESR1 remained relatively stable at approximately 130–135 nm^2^. β-sitosterol-SRC was approximately 245 nm^2^ and decreased slightly in the later stage. β-sitosterol-TNF decreased from approximately 265 nm^2^ to 250 nm^2^, indicating reduced solvent exposure in the later stage.

Rg analysis showed that β-sitosterol-AKT1 had the lowest Rg, approximately 1.43–1.48 nm, indicating the most compact overall structure among the AKT1 systems. The Rg of β-sitosterol-EGFR decreased from approximately 2.2 nm to 2.0 nm and then stabilised, suggesting gradual system contraction after binding. β-sitosterol-ESR1 remained stable at approximately 1.85 nm. The Rg of β-sitosterol-SRC was approximately 3.0–3.3 nm in the early stage and then decreased to approximately 2.7 nm, indicating marked system compression. β-sitosterol-TNF showed a small early peak and then stabilised at approximately 2.43–2.45 nm.

RMSD analysis showed that β-sitosterol-AKT1 fluctuated markedly in the early stage, with transient increases above approximately 2.0 nm within 0–20 ns, suggesting substantial initial conformational rearrangement. After 20 ns, the RMSD remained around 1.1 nm, with only a transient peak near 85 ns, indicating overall stability after equilibration. β-sitosterol-EGFR increased after the first 20 ns and stabilised at approximately 1.3–1.4 nm, suggesting a relatively stable but comparatively high conformational plateau. β-sitosterol-ESR1 showed multiple transitions, with higher RMSD intervals at approximately 30 ns and 65–80 ns, suggesting conformational conversion in this binding system. β-sitosterol-SRC showed a lower early RMSD and a plateau of approximately 1.3–1.5 nm after 60 ns, suggesting possible rearrangement of the binding pocket or structural domain. β-sitosterol-TNF remained relatively stable throughout the simulation, mainly at approximately 0.8–0.9 nm, and was one of the more stable curves in this group.

Hydrogen-bond analysis showed that β-sitosterol-AKT1 formed few hydrogen bonds, with only occasional counts of 1–2, indicating that its binding was not dependent on persistent hydrogen bonds. β-sitosterol-EGFR showed an increased hydrogen-bond frequency after 60 ns, mostly 1–2 bonds, suggesting the formation of some polar contacts in the later stage. β-sitosterol-ESR1 showed more hydrogen bonds in the later stage, reaching 3–4 near the end, but its RMSD also showed large transitions, indicating that increased hydrogen bonding did not necessarily correspond to the greatest overall stability. β-sitosterol-SRC formed hydrogen bonds more frequently, with 1–3 bonds, indicating that polar interactions existed in addition to hydrophobic effects. β-sitosterol-TNF formed few hydrogen bonds, suggesting that TNF binding was mainly driven by hydrophobic and van der Waals interactions. Overall, β-sitosterol did not appear to act through persistent hydrogen-bond dependence but was more likely to stabilise receptors through hydrophobic burial, van der Waals interactions and local conformational compression.

Figure 11E–I compares SASA, Rg, hydrogen-bond number, RMSD and RMSF changes for β-sitosterol and quercetin with AKT1.

SASA analysis showed that β-sitosterol-AKT1 fluctuated mainly within 78–86 nm^2^, with a transient increase around 55–65 ns. The SASA of quercetin-AKT1 was slightly higher at the early stage and approached 90 nm^2^ at 20–30 ns, then generally decreased and became similar to β-sitosterol-AKT1, indicating limited differences in solvent exposure between the two AKT1 systems.

Rg analysis showed that β-sitosterol-AKT1 mostly remained at approximately 1.42–1.47 nm, indicating a compact structure. The early Rg of quercetin-AKT1 was slightly higher, reaching approximately 1.50–1.52 nm, and gradually decreased to 1.42–1.45 nm after approximately 45 ns, suggesting a transition from a relatively loose to a compact state. The final Rg values of the two systems were similar, indicating stable overall folding.

RMSD analysis showed that β-sitosterol-AKT1 fluctuated strongly during the first 20 ns, reaching more than approximately 2.0 nm, and then stabilised at approximately 1.1 nm, with a transient peak near 85 ns. Quercetin-AKT1 showed a lower overall RMSD, mostly around 0.5 nm in the later stage, but frequent transient jumps appeared during 35–65 ns, suggesting staged adjustment of binding or local conformation.

Hydrogen-bond analysis showed that β-sitosterol-AKT1 formed very few hydrogen bonds, with only occasional 1–2 bonds. Quercetin-AKT1 exhibited abundant early hydrogen bonding, reaching up to five bonds, and 1–3 bonds were still intermittently observed in the later stage, indicating stronger polar interactions between quercetin and AKT1.

RMSF analysis showed highly similar RMSF curves for the two AKT1 complexes. Most residues were below 0.4 nm, with only the terminal residue region increasing markedly to approximately 0.9–1.0 nm, indicating that ligand differences mainly affected the binding region or terminal flexibility and had limited perturbation on the main AKT1 structure. The terminal peak was slightly higher for β-sitosterol, whereas quercetin showed slight fluctuations near some local peaks, but the overall difference was small.

Figure 11J–N compares RMSF, SASA, Rg, hydrogen-bond number and RMSD changes for emodin, β-sitosterol and kaempferol with EGFR.

SASA analysis showed that emodin-EGFR fluctuated between approximately 170 and 180 nm^2^, with a slight decrease in the later stage. β-sitosterol-EGFR was approximately 178–182 nm^2^ in the early stage and then decreased to approximately 168–172 nm^2^, indicating reduced solvent exposure. kaempferol-EGFR showed the highest early SASA, exceeding 190 nm^2^, and then decreased after 80 ns to become similar to the other two systems, suggesting a larger early exposed surface followed by contraction.

Rg analysis showed that emodin-EGFR gradually decreased from approximately 2.15–2.20 nm to approximately 2.05 nm, indicating increased compactness. β-sitosterol-EGFR decreased more markedly, reaching approximately 1.98–2.00 nm in the later stage and was the most compact of the three systems. Kaempferol-EGFR showed a high early Rg peak of approximately 2.5 nm, followed by a decrease and later convergence towards 2.0 nm, suggesting early conformational expansion and subsequent recompression.

RMSD analysis showed that emodin-EGFR fluctuated markedly during the first 20–30 ns and then stabilised at approximately 1.4–1.5 nm, with a slight increase near 85–90 ns followed by recovery, indicating basic stability in the later stage. β-sitosterol-EGFR increased from a low early RMSD to approximately 1.3–1.4 nm and remained relatively stable, suggesting entry into a stable plateau after conformational adjustment. Kaempferol-EGFR showed large early fluctuations, with a high peak near 35 ns; its RMSD decreased to approximately 1.05 nm during 60–80 ns and then increased again, suggesting clear conformational conversion or local binding-site rearrangement.

Hydrogen-bond analysis showed that emodin-EGFR exhibited obvious hydrogen bonding during 35–70 ns, reaching up to three bonds, indicating that emodin can form relatively stable polar contacts through hydroxyl and carbonyl groups. β-sitosterol-EGFR mainly formed 1–2 hydrogen bonds after 60 ns, consistent with its limited hydrogen-bonding sites. Kaempferol-EGFR formed hydrogen bonds earlier and more persistently, mostly 1–2 bonds; however, this did not translate into the strongest MM/PBSA binding free energy, indicating that hydrogen-bond number is not the sole determinant of binding free energy.

RMSF analysis showed that the three EGFR complexes generally had low RMSF values in the middle residue region, mostly below 0.5 nm, suggesting a stable main protein structure. N-terminal and C-terminal residues showed clearly increased fluctuations. Among them, kaempferol-EGFR had the highest C-terminal fluctuation, approaching or exceeding 2.0 nm, indicating that kaempferol may induce stronger flexibility changes in the terminal EGFR region. C-terminal fluctuations also increased in emodin-EGFR and β-sitosterol-EGFR but were generally lower than in kaempferol-EGFR.

#### 2.8.2. MM/PBSA Binding Free Energy and Residue Energy Decomposition

The contributions of individual MM/PBSA energy components to binding free energy are shown in Table 7. The ΔG values of all eight complexes were negative, indicating thermodynamically favourable binding trends in the selected trajectory segments. Table 7 reports ΔH and −TΔS separately, and the final ΔGbind values were calculated by including the entropy contribution according to ΔGbind = ΔH + (−TΔS). For example, for β-sitosterol-AKT1, ΔH was −27.44 kcal/mol and −TΔS was 8.56 kcal/mol, yielding a final ΔGbind of −18.88 kcal/mol. Based on the absolute binding free-energy values and the magnitude of negative ΔGbind, the binding stability ranked from strongest to weakest as follows: β-sitosterol-TNF (−28.29 kcal/mol), β-sitosterol-AKT1 (−18.88 kcal/mol), β-sitosterol-SRC (−14.14 kcal/mol), emodin-EGFR (−13.72 kcal/mol), β-sitosterol-ESR1 (−12.38 kcal/mol), kaempferol-EGFR (−9.73 kcal/mol), β-sitosterol-EGFR (−7.49 kcal/mol) and quercetin-AKT1 (−6.39 kcal/mol). Energy component analysis showed that the MM and SA terms were negative and were the major favourable contributors to binding, whereas PB and −TΔS terms were positive and unfavourable. The MM term of β-sitosterol-TNF reached −53.07 kcal/mol, substantially stronger than that of the other complexes, which primarily explained its optimal total ΔG. β-sitosterol-AKT1 and β-sitosterol-SRC also showed strong MM contributions. In contrast, quercetin-AKT1 had a weaker MM term and a PB solvation penalty; therefore, despite having more hydrogen bonds, its final ΔG remained relatively weak. This contrast indicates that the hydrogen-bond number alone is insufficient to determine the overall binding free energy, especially for hydrophobic ligands such as β-sitosterol.

**Figure 12:**
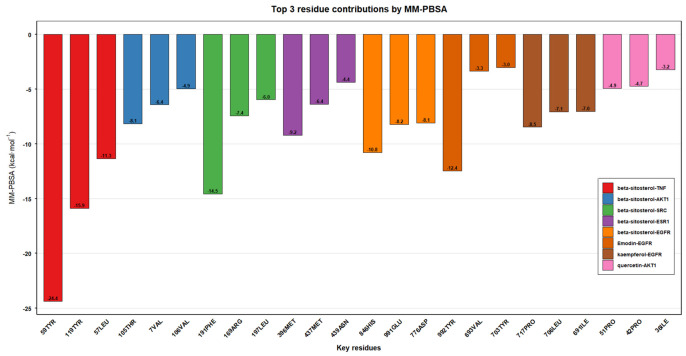
Energy decomposition plot of the top three residues contributing to MM/PBSA binding free energy in each complex.

The visualisation of the top three residues contributing to MM/PBSA binding free energy in each complex is shown in Figure 12. In the residue energy decomposition plot, the residue names on the x-axis correspond to the coloured bars; every three residues represent the top three contributors for each ligand–receptor complex.

Residue energy decomposition showed that Tyr39, Tyr119 and Leu57 in β-sitosterol-TNF contributed −24.4, −15.9 and −11.3 kcal/mol, respectively, representing the most prominent binding hotspots among all systems. Phe191 in β-sitosterol-SRC, His846 in β-sitosterol-EGFR and Tyr992 in emodin-EGFR also showed strong energy contributions. These findings suggest that aromatic and hydrophobic residues play important roles in the binding of PRD core compounds to key targets. In particular, the binding of β-sitosterol to TNF, AKT1 and SRC may constitute an important molecular basis for the effects of PRD on T2DM-associated inflammation and abnormal insulin signalling. Interpretations of the top three MM/PBSA contributing residues for each ligand–receptor complex are provided in Table 8.

## 3. Discussion

T2DM is a complex metabolic disease driven by insulin resistance, impaired pancreatic β-cell function, chronic low-grade inflammation, oxidative stress and lipid metabolic disorder. Single-target intervention often fails to comprehensively cover its multi-stage pathological processes, whereas traditional Chinese medicine formulas are characterised by multi-compound, multi-target and multi-pathway synergistic regulation. In this study, network pharmacology, molecular docking, molecular dynamics simulation and MM/PBSA binding free-energy analysis were integrated to generate a computational hypothesis for the potential mechanism by which PRD may act against T2DM. The results showed that the core compounds of PRD mainly included quercetin, kaempferol, formononetin, β-sitosterol and emodin, whereas the core targets mainly included AKT1, TP53, SRC, IL6, TNF, EGFR and ESR1. Enriched pathways mainly involved PI3K-Akt, MAPK, HIF-1, FoxO, mTOR, AGE-RAGE and TNF signalling. These results are consistent with the multi-pathway pharmacological effects previously observed by our group, but the novelty of the present work lies in prioritising candidate material-basis compounds and representative ligand–target interaction patterns, rather than rediscovering these pathways as entirely new PRD mechanisms.

Several core compounds identified in this study, including quercetin, kaempferol, luteolin and β-sitosterol, are widely distributed phytochemicals and are frequently reported in network pharmacology studies of herbal medicines. Therefore, their appearance should not be interpreted as unique to PRD at the single-compound level. The formulation specificity of PRD may instead arise from the co-occurrence of these compounds across its five component medicines, their relative positions in the PRD compound–target network and their convergence on inflammation, insulin signalling, oxidative stress and metabolic pathways. In this sense, the present study extends previous PRD pharmacodynamic studies by linking known phenotypic effects to a prioritised compound–target–pathway interaction network and to dynamic binding-stability hypotheses.

The PI3K-Akt signalling pathway is a central pathway in insulin signal transduction. After insulin binds to its receptor, the IRS/PI3K/AKT cascade is activated, thereby promoting glucose transport, glycogen synthesis and lipid metabolic homeostasis. AKT1 is a key node in this pathway and is closely related to insulin sensitivity and glucose metabolism regulation [9,23]. In this study, both PPI and KEGG analyses showed high network centrality of AKT1, suggesting that it may be an important target through which PRD acts against T2DM. MD results showed that β-sitosterol-AKT1 and quercetin-AKT1 formed relatively stable complexes. Quercetin-AKT1 had more hydrogen bonds, indicating stronger polar interactions with AKT1; however, MM/PBSA results showed that the binding free energy of β-sitosterol-AKT1 was lower, suggesting that hydrophobic and van der Waals interactions may contribute more substantially to binding stability in this system. Therefore, β-sitosterol and quercetin may regulate AKT1 through different interaction patterns and jointly participate in the modulation of insulin signalling.

The target and pathway overlap with established antidiabetic therapies further contextualise the predicted PRD network. Insulin and insulin analogues act through INSR-mediated PI3K-Akt, MAPK, FoxO and mTOR-related signalling; DPP-4 inhibitors and GLP-1 receptor agonists regulate downstream metabolic and β-cell-related pathways partly through incretin signalling; SGLT2 inhibitors primarily reduce renal glucose reabsorption but are also associated with modulation of inflammation, oxidative stress, AGE-RAGE-related injury and mTOR/AMPK-related processes [9,10,11,12,13,14,15,16,17,18,19,20,21]. The inclusion of DPP4, SLC5A2, PPARG, MGAM and INSR in the 483 predicted intersection targets suggests that the PRD target network overlaps with clinically validated antidiabetic pharmacology at selected nodes. Nevertheless, this overlap should be viewed as external support for network plausibility and not as direct evidence that PRD produces the same pharmacodynamic effects as approved drugs.

Chronic low-grade inflammation is an important pathological basis of insulin resistance in T2DM. Inflammatory mediators such as TNF and IL6 can activate NF-κB, JNK and MAPK signalling pathways, interfere with phosphorylation of insulin receptor substrates and weaken insulin signal transduction, thereby aggravating insulin resistance [24,25]. In this study, TNF and IL6 were located in the core region of the PPI network, suggesting that inflammatory response may be an important entry point for PRD in the treatment of T2DM. Molecular docking showed a low binding energy between β-sitosterol and TNF. Subsequent MD and MM/PBSA analyses showed that the MM/PBSA binding free energy of β-sitosterol-TNF was markedly lower than that of the other complexes. Residue energy decomposition identified Tyr39, Tyr119 and Leu57 as the main energetic contributors in this complex, suggesting that aromatic and hydrophobic residues participate in the formation of a stable hydrophobic binding interface. This finding supports the hypothesis that β-sitosterol may participate in the regulation of inflammatory targets in T2DM.

SRC and EGFR are important but pleiotropic nodes in tyrosine kinase-related signalling networks and participate in cell proliferation, inflammatory responses, oxidative stress and metabolic remodelling. They should therefore be interpreted as T2DM-related network hubs rather than T2DM-specific targets. Under diabetic conditions, inflammatory stimulation, advanced glycation end-product accumulation and oxidative stress can affect EGFR/SRC-related signalling, thereby contributing to tissue injury and metabolic disturbance [26,27]. This study showed that β-sitosterol-SRC, emodin-EGFR and β-sitosterol-EGFR all exhibited certain dynamic stability, among which β-sitosterol-SRC and emodin-EGFR had lower MM/PBSA binding free energies. In the EGFR systems, the Rg and SASA of β-sitosterol-EGFR decreased markedly in the later stage, suggesting increased compactness of the complex; however, its MM/PBSA binding free energy was weaker than that of emodin-EGFR. Thus, β-sitosterol-EGFR should be described as having a moderate predicted interaction in the PRD network, whereas emodin may provide a more favourable predicted EGFR interaction through polar contacts and aromatic interactions.

TP53 and EGFR are highly pleiotropic proteins, and their network centrality should not be overinterpreted as T2DM specificity. For TP53, hyperglycaemia, lipotoxicity, chronic inflammation and oxidative stress have been reported to activate p53-related stress responses, which may contribute to mitochondrial dysfunction, endoplasmic reticulum stress, cell-cycle arrest and apoptosis in pancreatic β cells, as well as inflammatory and senescence-like changes in adipose tissue [28,29]. For EGFR, EGF/EGFR signalling can connect growth-factor signalling with PI3K-Akt, MAPK/ERK and mTOR-related pathways, and has been implicated in β-cell survival, proliferation and compensatory responses under nutrient-excess conditions [30]. Therefore, PRD-related modulation of TP53- and EGFR-associated networks may be more appropriately interpreted as potential regulation of stress-response, β-cell compensatory and metabolic-inflammation pathways rather than direct targeting of diabetes-specific proteins.

Notably, β-sitosterol exhibited a prominent predicted multi-target binding tendency in this study [31]. Molecular docking results showed favourable docking scores between β-sitosterol and all seven core targets, and MD/MM-PBSA results further ranked β-sitosterol-TNF, β-sitosterol-AKT1 and β-sitosterol-SRC among the most favourable simulated complexes. Compared with polyphenolic compounds such as quercetin, kaempferol and emodin, β-sitosterol contains fewer groups capable of forming hydrogen bonds, but its hydrophobic steroidal skeleton more readily forms stable contacts with hydrophobic protein pockets or hydrophobic residue clusters. The apparent discrepancy between the abundant hydrogen bonds in quercetin-AKT1 and its relatively weak ΔGbind, and the few hydrogen bonds but strong ΔGbind of β-sitosterol-TNF, highlights that hydrogen-bond number alone is not a sufficient descriptor of binding stability. In these systems, hydrophobic burial, van der Waals interactions, aromatic/aliphatic residue contacts, desolvation penalties and entropy all contribute to the final binding free energy. Therefore, the action of β-sitosterol may not be characterised by persistent hydrogen-bond anchoring to a single target; rather, it may stabilise the binding interfaces of multiple inflammatory and metabolic targets through hydrophobic and van der Waals interactions. This characteristic is consistent with the multi-target regulatory features of traditional Chinese medicine formulas.

The docking and simulation workflow should also be interpreted cautiously. In the present study, molecular docking was used as a structure-based preliminary screening and conformation-generation step, and selected complexes were further evaluated by 100 ns MD simulation and MM/PBSA energy analysis. However, an independent validation step based on redocking of co-crystallised ligands and RMSD comparison was not performed for all receptors. Consequently, the docking poses should be considered computational binding hypotheses supported by subsequent dynamic-stability analysis, not experimentally validated ligand–receptor conformations. Future work should include co-crystallised ligand redocking or known inhibitor comparisons, such as EGFR-TKIs for EGFR, and should pair computational predictions with target phosphorylation, downstream AKT/ERK signalling and cell-function assays.

The absence of several docked complexes from the main MD/MM-PBSA ranking should not be interpreted as post hoc exclusion based on unfavourable results. The final eight complexes were selected before production MD to ensure mechanistic representativeness, cross-target comparison for β-sitosterol, within-target comparison for EGFR and AKT1, and feasible system parameterisation. TP53 had high centrality in the PPI network, and docking results suggested certain binding potential with β-sitosterol and other compounds. However, the selected TP53 protein structure contains metal ions and related coordination environments, which require dedicated treatment of metal-ion charges, coordination bonds and non-bonded parameters in classical MD simulations. To avoid artefacts from uncertain metal-centre parameterisation, TP53 was not included in the production MD/MM-PBSA analysis. IL6 was retained for docking visualisation and mechanism discussion, but its preliminary dynamic and binding-energy profiles did not provide a stronger representative comparison than the selected β-sitosterol-TNF, β-sitosterol-AKT1 and β-sitosterol-SRC systems.

This study has several limitations. First, network pharmacology results depend on database predictions, and disease targets and compound targets may include false positives, pleiotropic genes or overly broad coverage. The use of a GeneCards median relevance-score threshold and multi-database intersection can reduce but cannot eliminate this limitation. Second, the enrichment figures display −log10(*p*-value) as a visualised significance metric; adjusted *p*-values or FDR values should be exported and reported in future analyses when available. Third, molecular docking and MD simulations can only reflect potential binding capacity and dynamic stability between ligands and receptors; they cannot directly prove activation, inhibition or therapeutic efficacy at the cellular or organismal level. Fourth, MM/PBSA results are affected by force-field parameters, trajectory length, solvent model and entropy estimation method; thus, binding free energy should be interpreted as a relative trend rather than an absolute experimental binding constant. Finally, in vitro binding experiments, cellular functional assays and animal experiments have not yet been performed. Subsequent studies should verify the direct binding of β-sitosterol to TNF, AKT1, SRC and other targets using surface plasmon resonance, microscale thermophoresis, isothermal titration calorimetry or cellular thermal shift assays and should further validate the regulatory effects of PRD core compounds on insulin signalling and inflammatory pathways in high-glucose-induced HepG2, 3T3-L1, INS-1 and other cellular models and in T2DM animal models.

## 4. Materials and Methods

### 4.1. Screening of Active Compounds and Targets of PuRenDan

The Latin pharmaceutical names of each component medicine of PRD were searched in the Traditional Chinese Medicine Systems Pharmacology Database and Analysis Platform (TCMSP; https://www.tcmsp-e.com/tcmsp.php; accessed on 15 October 2025) and HERB 2.0 (http://47.92.70.12/; accessed on 16 October 2025): Ginseng Radix et Rhizoma, Momordicae Charantiae Fructus, Salviae Miltiorrhizae Radix et Rhizoma, Puerariae Lobatae Radix and Polygoni Multiflori Radix Praeparata. The corresponding botanical origins include *Panax ginseng* C.A.Mey., *Momordica charantia* L., *Salvia miltiorrhiza* Bunge, *Pueraria lobata* (Willd.) *Ohwi* and *Polygonum multiflorum* Thunb. Relevant phytochemical literature was also consulted to screen compounds from each component medicine of PRD. According to absorption, distribution, metabolism and excretion (ADME)-related criteria, active compounds were retrieved and screened in TCMSP using drug-likeness (DL) ≥ 0.18 and oral bioavailability (OB) ≥ 30% as thresholds, and additional compounds were supplemented from the literature [32,33]. These thresholds were selected because PRD is administered orally. OB reflects the likelihood of systemic exposure after oral administration, and DL evaluates the similarity of physicochemical and structural properties to known drugs. The OB ≥ 30% and DL ≥ 0.18 criteria are commonly used in TCMSP-based network pharmacology studies and support methodological comparability across herbal-medicine analyses [34,35,36]. Nevertheless, these thresholds are heuristic filters and may exclude some low-OB or low-DL compounds with potential local intestinal, metabolite-mediated or synergistic effects; therefore, the predicted active compounds require experimental validation.

Target proteins of the active compounds were retrieved from TCMSP. Reviewed human genes were downloaded from UniProt (https://www.uniprot.org/; accessed on 25 October 2025), and target proteins were converted into the corresponding gene names in Excel using the VLOOKUP function. SwissTargetPrediction (https://swisstargetprediction.ch/; accessed on 26 October 2025), PharmMapper Server (https://www.lilab-ecust.cn/pharmmapper/index.html; accessed on 26 October 2025) and related literature were then used to further supplement target proteins of the active compounds. Specifically, PubChem CIDs of the active compounds were searched in PubChem (https://pubchem.ncbi.nlm.nih.gov/; accessed on 26 October 2025), SDF structure files were downloaded and imported into SwissTargetPrediction and PharmMapper for target prediction [37,38].

### 4.2. Screening of T2DM-Associated Disease Targets

The disease module was selected in HERB 2.0 (http://47.92.70.12/; accessed on 10 November 2025), DrugBank (https://go.drugbank.com/; accessed on 10 November 2025), DisGeNET (https://disgenet.com/; accessed on 10 November 2025), the Human Phenotype Ontology (HPO; https://hpo.jax.org/; accessed on 12 November 2025), OMIM (https://www.omim.org/; accessed on 29 October 2025) and ClinPGx (https://www.clinpgx.org/; accessed on 12 November 2025), and the keyword “Type 2 Diabetes Mellitus” was searched. Targets appearing in at least two databases were selected as T2DM-associated disease targets. GeneCards (https://www.genecards.org/; accessed on 12 November 2025) was also searched, and targets with a relevance score above the median were selected as supplementary targets. The median GeneCards relevance-score threshold was used as a data-driven heuristic to avoid an arbitrary absolute cut-off while retaining sufficient disease-network coverage for downstream PPI and enrichment analyses, and similar median-based filtering has been used in recent network pharmacology studies [34,35,36]. Because this strategy may still retain weakly related genes, external pharmacological plausibility was further assessed by checking whether established T2DM drug targets were included in the final intersection set.

### 4.3. Screening of Compound–Disease Intersection Targets and Network Construction

The drug targets of PRD active compounds and T2DM-associated disease targets were uploaded to the Bioinformatics online platform (https://www.bioinformatics.com.cn/static/others/jvenn/example.html; accessed on 15 November 2025) to generate Venn diagrams and identify intersection targets, which were defined as active targets of PRD for the treatment of T2DM.

Network and type files describing the herb–compound–active target relationships were prepared and imported into Cytoscape 3.10.4 for network construction. To make the network sufficiently clear, some target series were processed and represented by asterisks [39]. The Analyze Network function was used to calculate degree, betweenness and closeness values of active compounds. The analysed data were imported into SPSS 27 for algorithmic scoring. Each topological parameter was standardised by selecting “save standardised scores as variables” in SPSS to generate ZDegree, ZBetweenness and ZCloseness. The comprehensive score was then calculated as Score = ZBetweenness + ZCloseness + ZDegree, and the top seven active compounds were selected as preliminary key compounds. Degree represents the number of direct connections of a node in the network and directly reflects its interaction frequency with other nodes; nodes with more connections are more likely to serve as core hubs. Betweenness reflects the intermediary role of a node in the shortest paths between other nodes; the more frequently this bridging role occurs, the stronger the potential ability of the node to regulate information or signal transmission in the network. Closeness reflects how readily a node can reach all other nodes in the network; a higher value suggests a more rapid role in processes such as information diffusion. Negative comprehensive scores indicate below-average centrality in the analysed node set after standardisation and do not represent negative biological activity.

### 4.4. PPI Network Construction and Core Target Screening

Drug targets of PRD active compounds and T2DM-associated disease targets were uploaded to the STRING database (https://cn.string-db.org/ [40,41]; accessed on 17 November 2025). “Multiple proteins” was selected, the species was set to Homo sapiens, and the confidence score was set to 0.4. After screening, PPI network data were imported into Cytoscape 3.10.4. The Analyze Network function was used to calculate degree, betweenness and closeness values. These data were imported into SPSS 27 for algorithmic scoring. As described above, ZDegree, ZBetweenness and ZCloseness were generated by Z-score standardisation, and the final score was calculated as Score = ZBetweenness + ZCloseness + ZDegree. The top 15 targets were selected as preliminary key targets according to this comprehensive topological score.

### 4.5. GO and KEGG Enrichment Analyses

Drug targets of PRD active compounds and T2DM-associated disease targets were uploaded to Metascape (https://metascape.org/gp/#/main/step1; accessed on 20 November 2025), and the species was set to Homo sapiens. Biological process (BP), cellular component (CC) and molecular function (MF) were selected for GO enrichment analysis. After the corresponding results were obtained, the top 10 enriched terms according to −log10 (*p*-value) were uploaded to the Bioinformatics online platform for visualisation [42]. In the figures and supplementary enrichment tables, the column labelled as the visual significance metric represents −log10 (*p*-value), not the raw *p*-value itself.

KEGG pathways were selected for enrichment analysis in Metascape. The top 25 pathways ranked by −log10 (*p*-value) were uploaded to the Bioinformatics online platform for visualisation, and a target-pathway GO chord plot was generated. KEGG analysis data were imported into Cytoscape 3.10.4 to construct a compound–target–pathway network for the top 25 pathways. Degree, betweenness and closeness analyses were performed using the Analyze Network function. The resulting data were imported into SPSS 27 for scoring using the same standardised additive formula described above; compounds and targets were ranked according to Score = ZBetweenness + ZCloseness + ZDegree. The top seven compounds in the compound–target–pathway network were defined as KEGG core compounds. These were analysed together with the preliminary core compounds described above to determine the final credible key compounds and key targets.

### 4.6. Molecular Docking Validation

The mol2 structures of the top five key compounds, quercetin, kaempferol, formononetin, β-sitosterol and emodin, were downloaded from TCMSP (https://www.tcmsp-e.com/tcmsp.php; accessed on 15 March 2026). OBGUI software and batch commands were used to process the mol2 files in batches for energy minimisation and conversion into pdbqt format.

The 3D protein structure PDB files of the top seven key targets were downloaded from the RCSB PDB database (RCSB.org; https://www.rcsb.org/; accessed on 17 March 2026). Detailed information on the 3D protein structures is shown in Table 9. Ligands and water molecules were removed using PyMOL3.1.8, and the files were imported into AutoDockTools1.1.2 for hydrogen addition and charge assignment. The processed structures were designated as receptors and exported in pdbqt format.

The pdbqt files of the 3D proteins were imported into AutoDockTools1.1.2. Docking grids were set to cover the selected receptor binding regions and, when available, annotated or co-crystallised ligand-binding cavities; for targets without a clearly defined small-molecule pocket, the docking box was expanded to include the relevant accessible binding region of the protein. Vina and batch processing commands were prepared for pairwise molecular docking between the five ligands and seven receptors [50]. Docking parameters were set as follows: exhaustiveness = 16, num_modes = 20 and energy_range = 5. After batch docking, binding energies and output.pdbqt files were obtained. The conformation with the largest absolute binding-energy value was selected as the optimal docking conformation. The optimal docking conformation data were imported into R for heatmap visualisation, and output.pdbqt files with favourable ligand–receptor binding were visualised using PyMOL3.1.8 and LigPlus2.3.1. Each predicted pose was inspected to ensure that the ligand was located within the predefined binding region and formed plausible polar or hydrophobic contacts with pocket residues. Because co-crystallised ligand redocking and RMSD validation were not performed for every receptor, the docking results were used only as preliminary structure-based predictions and as starting conformations for subsequent MD and MM/PBSA analyses.

### 4.7. Molecular Dynamics Simulation

#### 4.7.1. MD Simulation

Based on molecular docking results, ligand–receptor complexes with favourable and representative binding conformations were selected for MD simulation before production MD was conducted. The eight selected complexes were TNF-β-sitosterol, AKT1-β-sitosterol, ESR1-β-sitosterol, EGFR-β-sitosterol, SRC-β-sitosterol, EGFR-emodin, EGFR-kaempferol and AKT1-quercetin. TNF-β-sitosterol, AKT1-β-sitosterol, ESR1-β-sitosterol, EGFR-β-sitosterol and SRC-β-sitosterol were used to compare the same key compound across different core targets; EGFR-β-sitosterol, EGFR-emodin and EGFR-kaempferol were used to compare different representative compounds acting on EGFR; and AKT1-β-sitosterol and AKT1-quercetin were used to compare different compounds acting on AKT1. Therefore, the selection was based on network centrality, docking affinity, compound representativeness, cross-comparison value and parameterisation feasibility, not on post hoc exclusion of complexes with unfavourable RMSD, RMSF or MM/PBSA results. The optimal docking conformations generated by AutoDock Vina1.1.2 and the corresponding receptor structures were imported into PyMOL3.1.8, and receptor protein and small-molecule ligand structures were extracted separately. Receptor proteins were saved in PDB format. After removal of water molecules and non-target organic molecules, small-molecule ligands were hydrogenated and saved in MOL2 format. Missing atoms in receptor proteins were completed using Swiss-PdbViewer (SPDBV) before subsequent simulation.

Small-molecule ligand topologies were generated using Sobtop. GAFF atom types were first assigned on the basis of MOL2 structures. Missing parameters were supplemented and predicted using built-in Sobtop rules, and GROMACS-compatible ligand topology (.itp) and coordinate (.gro) files were then generated. Protein topologies were generated using the pdb2gmx module in GROMACS 2023.2. The CHARMM36 all-atom force field and CHARMM-modified TIP3P water model were selected. Protein and ligand coordinate and topology files were then merged using a custom Python script to construct the protein–ligand complex systems.

MD simulations were performed using GROMACS 2023.2. Each complex was first placed in a cubic periodic box with a minimum distance of 1.0 nm between the complex surface and the box boundary. Water molecules were then added, and Na+ and Cl- ions were introduced to neutralise the system charge while setting the ion concentration to 0.15 mol/L. After system construction, energy minimisation was performed using the steepest descent algorithm to remove unreasonable atomic contacts. Following energy minimisation, position restraints were applied to the ligand, and NVT and NPT equilibration simulations were performed sequentially to stabilise the system temperature and pressure. Each production MD simulation was performed for 100 ns. Production MD simulations were conducted under the NPT ensemble to obtain dynamic trajectories of the complex systems.

#### 4.7.2. MD Trajectory Analysis

After simulation, complex trajectories were analysed using built-in GROMACS tools. Root mean square deviation (RMSD) was used to evaluate overall conformational stability of the complexes; root mean square fluctuation (RMSF) was used to analyse residue-level protein flexibility; radius of gyration (Rg) was used to evaluate structural compactness; the number of protein–ligand hydrogen bonds was used to analyse polar interactions between ligands and receptors; and solvent accessible surface area (SASA) was used to evaluate surface exposure. RMSD analysis was performed separately for the protein backbone, ligand, receptor protein and protein–ligand complex. RMSF, Rg, hydrogen-bond and SASA analyses were used to characterise local protein flexibility, overall compactness, intermolecular interactions and solvent exposure, respectively. RMSF was used only for comparisons of different ligands acting on the same target, such as AKT1 and EGFR, because different receptor proteins vary substantially in amino acid length, residue numbering, domain composition and flexible regions. Direct comparison of RMSF curves among different receptors could therefore confound interpretation; for cross-receptor comparison of β-sitosterol, global indicators more suitable for different proteins, including RMSD, Rg, SASA and hydrogen-bond number, were used. All trajectory data were organised and visualised using Python scripts and DuIvyTools.

#### 4.7.3. MM/PBSA Binding Free-Energy Calculation and Residue Energy Decomposition

To further evaluate the binding stability of ligand–receptor complexes, MM/PBSA calculations based on GROMACS trajectories were performed to estimate binding free energy. Production MD trajectories were first corrected for periodic boundary conditions, centred and repaired for molecular integrity. The final 20 ns stable trajectory segment was then extracted for MM/PBSA analysis. MM/PBSA calculations were performed using the gmx_mmpbsa.bsh script. APBS was used to solve the polar solvation energy, the ion strength was set to 0.15 mol/L and the Debye–Hückel model was used as the dielectric model. The calculation output included enthalpy (ΔH), entropy contribution (−TΔS) and final binding free energy (ΔGbind), and Table 7 reports −TΔS as a separate component to clarify that entropy was included in the final ΔGbind values. Binding free energy was calculated as follows:

ΔGbind = Gcomplex − Greceptor − Gligand = ΔEMM + ΔGPB + ΔGSA − TΔS.

In this equation, ΔEMM represents molecular mechanics energy, including van der Waals and electrostatic interactions; ΔGPB represents polar solvation energy; ΔGSA represents non-polar solvation energy; and −TΔS represents the entropic contribution. Finally, residue energy decomposition was performed based on the MM/PBSA results to screen the top three key residues contributing to binding free energy in each complex, followed by visualisation [51].

## 5. Conclusions

This study systematically investigated the potential mechanisms by which PRD may act against T2DM using network pharmacology, molecular docking, MD simulation and MM/PBSA binding free-energy analysis. The results suggest that PRD may act on disease-related network hubs, including AKT1, TNF, SRC, EGFR, ESR1, IL6 and TP53 through candidate compounds such as quercetin, kaempferol, formononetin, β-sitosterol and emodin, and may regulate key T2DM-related pathological processes, including insulin resistance, chronic inflammation, oxidative stress and glucose–lipid metabolic abnormalities, via the PI3K-Akt, MAPK, HIF-1, FoxO, mTOR, AGE-RAGE and TNF signalling pathways.

Molecular simulation further suggested that β-sitosterol has a favourable predicted multi-target binding tendency. Among the simulated complexes, β-sitosterol-TNF, β-sitosterol-AKT1 and β-sitosterol-SRC displayed favourable dynamic stability and low predicted MM/PBSA binding free energies. Residue energy decomposition suggested that Tyr39, Tyr119, Leu57, Thr105, Val7 and Phe191 may be important binding hotspots maintaining complex stability. The binding of β-sitosterol to core targets mainly depended on hydrophobic interactions, aromatic residue interactions and van der Waals interactions rather than persistent hydrogen bonds. Complexes such as emodin-EGFR, kaempferol-EGFR and quercetin-AKT1 also showed certain predicted binding stability, suggesting that PRD may exert synergistic regulation through multiple representative compounds.

In summary, the potential mechanism of PRD against T2DM may be associated with molecular networks involving β-sitosterol-TNF/AKT1/SRC and emodin/kaempferol/quercetin-EGFR/AKT1. Its core effects may involve anti-inflammatory activity, improvement of insulin signal transduction and regulation of metabolism-related pathways. This study provides computational biology evidence and prioritised hypotheses for the material basis and molecular mechanisms of PRD in the treatment of T2DM, but these predictions require further validation by in vitro binding assays, cellular functional experiments and animal studies before therapeutic efficacy or direct target modulation can be confirmed.

## Figures and Tables

**Table 1:** Shared active compounds among the component medicines of PuRenDan.

ID	Active Compound	OB (%)	DL	Source
A1	luteolin	36.16	0.25	*Momordicae Charantiae Fructus*; *Salviae Miltiorrhizae Radix et Rhizoma*
B1	β-sitosterol	36.91	0.75	*Ginseng Radix et Rhizoma*; *Puerariae Lobatae Radix*; *Polygoni Multiflori Radix Praeparata*
C1	quercetin	46.43	0.28	*Momordicae Charantiae Fructus*; *Polygoni Multiflori Radix Praeparata*
D1	kaempferol	41.88	0.24	*Ginseng Radix et Rhizoma*; *Momordicae Charantiae Fructus*; *Polygoni Multiflori Radix Praeparata*

OB: oral bioavailability; DL: drug likeness.

**Table 2:** Seven preliminarily screened core compounds of PuRenDan.

ID	Core Compound	Betweenness	Closeness	Degree	Score	Source
D1	kaempferol	0.13	0.52	225	19.72	*Ginseng Radix et Rhizoma; Momordicae Charantiae Fructus*; *Polygoni Multiflori Radix Praeparata*
C1	quercetin	0.15	0.53	148	18.07	*Momordicae Charantiae Fructus*; *Polygoni Multiflori Radix Praeparata*
GG2	formononetin	0.05	0.44	147	10.10	*Puerariae Lobatae Radix*
B1	β-sitosterol	0.05	0.43	114	8.51	*Ginseng Radix et Rhizoma; Puerariae Lobatae Radix*; *Polygoni Multiflori Radix Praeparata*
GG3	3′-methoxydaidzein	0.04	0.43	111	7.45	*Puerariae Lobatae Radix*
ZSW8	emodin	0.04	0.44	63	6.27	*Polygoni Multiflori Radix Praeparata*
A1	luteolin	0.04	0.45	46	6.11	*Momordicae Charantiae Fructus*; *Salviae Miltiorrhizae Radix et Rhizoma*

**Table 3:** Core targets of PuRenDan.

Core Target	Betweenness	Closeness	Degree	Score
AKT1	0.04	0.69	267	15.28
TP53	0.04	0.68	258	13.88
SRC	0.04	0.63	210	12.91
IL6	0.03	0.67	251	12.73
TNF	0.03	0.67	251	12.72
EGFR	0.03	0.65	224	10.92
ESR1	0.02	0.63	208	9.88
CTNNB1	0.02	0.64	212	9.16
IL1B	0.02	0.64	215	8.93
MYC	0.02	0.64	213	8.75
CASP3	0.02	0.64	218	8.36
PTGS2	0.02	0.61	182	8.33
STAT3	0.02	0.64	214	8.20
HSP90AA1	0.02	0.62	192	8.11
PPARG	0.02	0.61	180	7.94

**Table 4:** Seven core compounds screened by KEGG analysis of PuRenDan.

ID	Core Compound	Betweenness	Closeness	Degree	Score	Source
C1	quercetin	0.12	0.46	107	18.21	*Momordicae Charantiae Fructus*; *Polygoni Multiflori Radix Praeparata*
D1	kaempferol	0.05	0.42	121	12.68	*Ginseng Radix et Rhizoma*; *Momordicae Charantiae Fructus*; *Polygoni Multiflori Radix Praeparata*
RS15	inermin	0.04	0.40	38	6.18	*Ginseng Radix et Rhizoma*
ZSW8	emodin	0.03	0.40	43	5.63	*Polygoni Multiflori Radix Praeparata*
A1	luteolin	0.02	0.40	40	4.47	*Momordicae Charantiae Fructus*; *Salviae Miltiorrhizae Radix et Rhizoma*
ZSW12	physcion	0.02	0.40	34	4.25	*Polygoni Multiflori Radix Praeparata*
GG2	formononetin	0.02	0.38	45	4.16	*Puerariae Lobatae Radix*

**Table 5:** Seven final core compounds of PuRenDan.

ID	Core Compound	Initial Score	Analysis Score	Final Score	Source
C1	quercetin	18.07	18.21	3.92	*Momordicae Charantiae Fructus*; *Polygoni Multiflori Radix Praeparata*
D1	kaempferol	19.72	12.68	3.15	*Ginseng Radix et Rhizoma*; *Momordicae Charantiae Fructus; Polygoni Multiflori Radix Praeparata*
GG2	formononetin	10.10	4.16	−0.21	*Puerariae Lobatae Radix*
B1	β-sitosterol	8.51	3.76	−0.57	*Ginseng Radix et Rhizoma*; *Puerariae Lobatae Radix; Polygoni Multiflori Radix Praeparata*
ZSW8	emodin	6.27	5.63	−0.60	*Polygoni Multiflori Radix Praeparata*
RS15	inermin	5.33	6.18	−0.67	*Ginseng Radix et Rhizoma*
A1	luteolin	6.11	4.47	−0.86	*Momordicae Charantiae Fructus*; *Salviae Miltiorrhizae Radix et Rhizoma*

**Table 6:** Molecular docking binding energies of core compounds with key targets.

Ligand	TNF (2AZ5)	SRC (1Y57)	EGFR (1M17)	IL6 (4J4L)	ESR1 (1SJ0)	TP53 (1DT7)	AKT1 (2UZR)
β-sitosterol	−9.9	−9.5	−9.3	−8.5	−8.4	−8.4	−7.4
emodin	−7.7	−8.5	−8.8	−7.6	−8.2	−7.8	−6.8
formononetin	−7.8	−7.5	−7.7	−7.0	−7.7	−7.8	−6.0
kaempferol	−7.9	−8.7	−8.4	−7.7	−8.1	−7.4	−6.5
quercetin	−7.6	−8.6	−8.8	−7.5	−7.2	−7.4	−6.5

Binding energies are expressed in kcal/mol.

**Table 7:** Contributions of MM/PBSA energy components to the binding free energies of the complexes.

Ligand–Receptor Complex	ΔGSA	ΔGPB	ΔEMM	ΔH	−TΔS	ΔGbind
β-sitosterol-TNF	−5.26 ± 0.12	13.33 ± 0.92	−53.07 ± 2.67	−44.99 ± 2.61	16.70	−28.29
β-sitosterol-AKT1	−3.84 ± 0.44	9.11 ± 1.17	−32.72 ± 5.28	−27.44 ± 5.37	8.56	−18.88
β-sitosterol-SRC	−3.59 ± 0.36	9.96 ± 3.51	−29.05 ± 3.52	−22.68 ± 1.32	8.54	−14.14
β-sitosterol-ESR1	−3.61 ± 0.35	13.24 ± 2.57	−30.33 ± 4.64	−20.70 ± 3.53	8.32	−12.38
β-sitosterol-EGFR	−3.07 ± 0.45	10.67 ± 2.61	−28.54 ± 5.44	−20.94 ± 4.12	13.45	−7.49
emodin-EGFR	−3.83 ± 0.33	14.07 ± 2.93	−27.56 ± 2.54	−17.32 ± 2.24	3.60	−13.72
kaempferol-EGFR	−2.98 ± 0.29	6.74 ± 2.28	−20.57 ± 4.00	−16.82 ± 2.68	7.09	−9.73
quercetin-AKT1	−2.86 ± 0.33	11.03 ± 2.91	−20.58 ± 3.42	−12.41 ± 3.87	6.02	−6.39

All energy terms are expressed in kcal/mol.

**Table 8:** Interpretation of the top three MM/PBSA contributing residues.

Complex	Top Three Key Residues and Contributions (kcal/mol)	Interpretation
β-sitosterol-TNF	Tyr39 (−24.4); Tyr119 (−15.9); Leu57 (−11.3)	The strongest hotspot combination; aromatic and hydrophobic residues dominate, explaining the optimal ΔG.
β-sitosterol-AKT1	Thr105 (−8.1); Val7 (−6.4); Val106 (−4.9)	Val residues indicate an important role for hydrophobic interactions, while Thr105 may provide local polar anchoring.
β-sitosterol-SRC	Phe191 (−14.5); Arg169 (−7.4); Leu197 (−6.0)	Phe/Leu hydrophobic contacts and aromatic stacking contribute prominently, supporting strong β-sitosterol-SRC binding.
β-sitosterol-ESR1	Met396 (−9.2); Met437 (−6.4); Asn439 (−4.4)	Met hydrophobic residues dominate, and Asn may contribute to polar stabilisation.
β-sitosterol-EGFR	His846 (−10.8); Glu991 (−8.2); Asp776 (−8.1)	Individual residue contributions are not weak, but the total ΔG is affected by PB and entropic penalties and is inferior to emodin-EGFR.
emodin-EGFR	Tyr992 (−12.4); Val693 (−3.3); Tyr703 (−3.0)	Tyr992 is the central hotspot, combining hydrogen bonding and aromatic interactions, which explains the energetic advantage of emodin-EGFR.
kaempferol-EGFR	Pro717 (−8.5); Leu706 (−7.1); Ile691 (−7.0)	A balanced hydrophobic residue cluster contribution is consistent with the moderately strong binding of kaempferol-EGFR.
quercetin-AKT1	Pro51 (−4.9); Pro42 (−4.7); Ile36 (−3.2)	Single-residue contributions are relatively weak; despite multiple hydrogen bonds, strong energetic hotspots are insufficient.

**Table 9:** Three-dimensional protein structure information for key targets.

Key Target	PDB	Structure Determination Method	Resolution	Reference
AKT1	2UZR	X-ray diffraction	1.94 Å	[43]
IL6	4J4L	X-ray diffraction	2.30 Å	[44]
TNF	2AZ5	X-ray diffraction	2.10 Å	[45]
EGFR	1M17	X-ray diffraction	2.60 Å	[46]
ESR1	1SJ0	X-ray diffraction	1.90 Å	[47]
TP53	1DT7	Solution NMR	40 submitted conformers	[48]
SRC	1Y57	X-ray diffraction	1.91 Å	[49]

## Data Availability

The datasets analysed in this study were obtained from public databases cited in the Materials and Methods section and from the literature. Additional data are available from the corresponding authors upon reasonable request.

## Supplementary Materials

The following supporting information can be downloaded at: https://www.mdpi.com/article/10.3390/ph19071107/s1, Table S1: Screened Components and Targets of Purendan; Table S2: The information of the top 10 GO-BP pathways obtained through screening; Table S3: The information of the top 10 GO-CC pathways obtained through screening; Table S4: The information of the top 10 GO-MF pathways obtained through screening; Table S5: The information of the top 25 KEGG pathways obtained through screening.

## Abbreviations

The following abbreviations are used in this manuscript:

ADME	Absorption, distribution, metabolism and excretion
AGE-RAGE	Advanced glycation end-product-receptor for advanced glycation end-product
BP	Biological process
CC	Cellular component
DL	Drug-likeness
GO	Gene Ontology
HPO	Human Phenotype Ontology
KEGG	Kyoto Encyclopedia of Genes and Genomes
MD	Molecular dynamics
MF	Molecular function
MM/PBSA	Molecular mechanics/Poisson–Boltzmann surface area
OB	Oral bioavailability
PDB	Protein Data Bank
PPI	Protein–protein interaction
PRD	PuRenDan
Rg	Radius of gyration
RMSD	Root mean square deviation
RMSF	Root mean square fluctuation
SASA	Solvent accessible surface area
T2DM	Type 2 diabetes mellitus
