# Supplementary material for: Material Basis and Mechanisms of Action of PuRenDan in the Treatment of Type 2 Diabetes Mellitus: An Integrated Network Pharmacology and Molecular Simulation Study
Source: Pharmaceuticals (Basel). 2026 Jul 17;19(7):1107. doi: 10.3390/ph19071107 (PMC13415839; doi:10.3390/ph19071107)
Supplement: Supplementary file 1 [file pharmaceuticals-19-01107-s001.zip › pharmaceuticals-4348367-supplementary.pdf]

Supplementary Table

Table S1. Screened Components and Targets of Purendan

Drug	Ingredient	Symbols
GINSENGRADIXETRHIZOMA	Diop,Stigmasterol,beta-sitosterol,Inermin,kaempferol,Chrysanthemaxanthin,Aposiopolamine,Celabenzine,Deoxyharringtonine,Dianthramine,arachidonate,FrutinoneA,ginsenosiderh2,Ginsenoside-Rh4_qt,Girinimbin,GomisinB,malkangunin,Panaxadiol,suchilactone,alexandrin_qt,ginsenosideRg5_qt,Fumarine	CYP1B1,NR1H3,AHR,ALOX5,UGT1A8,SREBF2,CYP7A1,ALOX12,CDK1,UGT1A7,TRPV4,RPS6KA3,KIAA0101,ABCA1,IL10,PTGS1,DHCR24,BCL2A1,SREBF1,HRH1,ABCG5,ICAM1,TBXA2R,NR1I2,CASP3,UGT3A1,ABCG8,PRKCA,UGT1A3,F2,WDTC1,ABCB11,APOE,PPIG,PLA2G1B,PTGS2,IL8,SLCO1B1,UGT1A9,TNF,CA2,PPARD,FABP4,NPC1L1,ABCC1,XDH,ACHE,TYR,PPARG,CA4,HSD17B1,NOX4,ABCB1,ESRRA,PPARA,HSD17B2,FLT3,ABCG2,CA12,CA7,ADORA1,AR,AKR1B1,PSMD3,HTR2A,HTR3A,AHSA1,ADH1C,AKR1C3,GUSBP1,ADRA1A,ADRA1B,ADRA1D,ADRA2A,MAOA,MAOB,SLPI,BAX,BCL2,ADRB1,ADRB2,KCNMA1,CALM,CASP1,CASP8,CASP9,CDC2,PDEA,CTRB1,F7,F10,CYP1A1,CYP1A2,CYP3A4,CYP101A1,OPRD1,DPP4,TOP2,DRD1,MAP2K4,SELE,GABRA1,GABRA2,GABRA3,GABRA5,NR3C1,GSTM1,GSTM2,GSTP1,HSP90,HMOX1,HAS2,IGHG1,IKBKB,INSR,IFNG,IL1B,MMP1,LTA4H,MAP2,NR3C2,MAPK8,PRKACA,PTPN1,CHRM1,CHRM2,CHRM3,CHRM4,CHRM5,OPRM1,CHRNA7,CHRNA2,NFKBIA,NOS2,NOS3,NCOA1,NCOA2,NR1I3,PRXC1A,PIK3CG,KCNH2,PGR,PSMG1,AKT1,RXRA,RXRG,Ppp3cb,PON1,STAT1,SCN5A,SLC6A3,SLC6A2,SLC6A4,SLC2A4,TFAP4,PRSS1,DIO1,Pde4,PLAU,VCAM1,KDR,CACNA1S,ME2,ME3,NA,cobT,NOS1,PTEN,PTGER3,ACP3,RUNX1T1,SRC,RB1,RHO,rsbQ,Slc22a5,TEP1,PRSS3,ALOX15,MAPKAPK2,CSNK1G1,RPS6KA1,ROCK1,MAPK1,ERN1,RET,DAO,GRM4,HDAC2,TTR,DGAT1,HDAC4,GRK2,ALPG,PLAA,HSD17B3,MTOR,PIK3CD,PIK3CB,PIK3CA,PGF,VEGFA,CHEK1,WEE1,TUBB1,TUBB3,CCND3CCND1CDK4CCND2,ABL1,RAF1,JAK3,LCK,CDK2,PRKDC,HCK,PI4KB,PIM2,BRAF,EPHB4,MMP8,CXCR1,PIM3,TYMS,GUSB,CA14,PARP1,MAP3K8,NEK1,CDK5R1CDK5,CCNB3CDK1CCNB1CCNB2,GSK3B,COMT,ADORA2B,HDAC5,HDAC7,MKNK1,DRD4,OPRK1,CCR5,EPHX2,EGFR,CHRNA4CHRNB2,CTSB,PRCP,THRB,MC4R,ACACA,ACACB,CAPN1,P2RX7,EP300,TACR1,HSD11B1,DRD2,DRD3,CCR3,PGGT1BFNTA,SIGMAR1,ITGA2BITGB3,MLYCD,LGMN,GABRB3GABRG2GABRA1,GABRB3GABRG2GABRA5,PDE10A,TNKS2,TNKS,CYP27A1,SCD,L3MBTL3,CYP11B2,PARP3,GPBAR1,PARP4,L3MBTL1,BAZ2B,CECR2,BAZ2A,CCR1,CREBBP,UTS2R,TACR2,PPIA,PLA2G2A,SYK,PDE5A,APP,MET,PDE4D,MAPK13,GRM1,TGFBR1,AURKB,AURKA,CFD,PSMB5,MAPK14,CRHR1,CHRNB1CHRNA1CHRNGCHRND,KCNK3,NTRK1,SLC6A15,H1F0,CPA1,DUT,HNMT,CYP11B1,PANK3,AKR1C2,CTSV,CTSL,AKR1C1,CA1,CA9,FOLH1,MPO,FFAR1,FABP3,CNR1,FAAH,TERT,FABP5,FABP1,TOP1,PTGES,PTGER2,PTGER1,PTPN11,CDC25A,PTGDR2,RARB,POLB,RXRB,RARG,RARA,TP53,SLC22A12,PTGER4,MDM2,ESR2,PTPN2,HSD11B2,CYP26B1,CYP26A1,TBXAS1,FABP2,HMGCR,PTGIR,PLA2G4A,AKR1B10,FFAR4,ENPP2,PTGES2,LTB4R,GLRA1,PRKCH,OXER1,CDC25B,NR

		0B2,TRPA1,DAGLA,PSEN2PSENENNCSTNAPH1APSEN1APH1B,AGTR1,GRM2,CNR2,SLC16A1,TRPM8,PDE4<br>A,HNF4A,DAGLB,TRPV1,MME,RBP4,LDHA,ESR1,PTGDR,ITGALICAM1ITGB2,EDNRA,S1PR4,RORC,PTPRF,<br>DHODH,PDE4B,CCKBR,FNTAFNTB,CMA1,CTSG,S1PR1,ALOX5AP,METAP1,PRKAG1PRKAB1PRKAA2,PLA2<br>G10,ACE,ACP1,CES2,CPT1A,NFKB1,CA5B,CA5A,CA13,CA6,CA3,PIK3CDPIK3R1,AGPAT2,TRAP1,HSP90B1,P<br>DE8B,HSP90AA1,ALPL,MAPK10,MAPK9,DYRK1A,BCHE,CLK4,HTR2B,HTR2C,HTR6,CLK2,DYRK3,SAE1UB<br>A2,TACR3,HRH2,ADRB3,ADORA2A,ADORA3,OXTR,NPY5R,ADRA2C,ADRA2B,TAAR1,IDH1,PPP1CA,GPR55<br>,FLT1,PIM1,ERBB2,PFKFB3,TSPO,LIPE,QPCT,GABRB3GABRA3GABRG2,GABRA2GABRB3GABRG2,VCP,NR<br>1H4,GABRG2GABRB3GABRA6,GABRB3GABRA4GABRG2,PDGFRB,FGFR1,HCRTR2,HCRTR1,AVPR2,EPHX1<br>,ELANE,P2RY1,DYRK2,PTAFR,ALK,NR1H2,HRH3,RORB,RORA,GRIN1GRIN2B,HTR1A,PDE7A,MGLL,DYRK<br>1B,MTNR1B,CLK1,GSK3A,PIK3CAPIK3R1,CYP17A1,PDE2A,NEK2,CCND1CDK4,FLT4,IGF1R,PDGFRA,CDK2<br>CCNA1CCNA2,PTK2,ROCK2,PLK4,TEK,LRRK2,JAK1,JAK2,DCTPP1,GABRA1GABRB2GABRG2,PKM,PORCN<br>,SLC5A2,ATP4BATP4A,TTK,TNNI3K,GRK3,PLK1,IRAK4,GRK5,SLC9A1,ASAH1,CHUK,TYK2,MTNR1A,CSNK<br>1A1,CSNK1D,PTK2B,CLK3,PDGFRAPDGFRB,F3,HTR7,CDC7,PARP2,TRHR,CYP19A1,MKNK2,CCNE1CDK2,P<br>DPK1,DUSP3,DRD5

FRUCTUS MOMORDICAE CHARANTIAE	quercetin,ellagic acid,apigenin,luteolin,Vemurafenib,Sorafenib,gallocatechin gallate,myricetin,kaempferol,biochanin a,ascorbic acid,3-(4-Hydroxyphenyl)propionic acid,L-citrulline,charantagenin D aglycone,Momordicoside C aglycone,momordicoside F1 aglycone,Momordicoside G aglycone,Momordicine I,Momordicoside L aglycone,(+)-Tirucallol,kuguacin C,gadoleic acid,goyaglycoside a aglycone,goyaglycoside b aglycone,goyaglycoside d aglycone,karaviloside II aglycone,Momordicoside A aglycone,Momordicoside B aglycone,karavilagenin A,Momordicoside K aglycone,(3beta,5alpha,24S)-Stigmasta-7,25-dien-3-ol,Diosgenin,goyaglycoside c aglycone,kuguaglycoside G aglycone,momordicoside D aglycone,karavilagenin E,kuguacin J,Momordicoside I F2 aglycone,naringin aglycone,linoleic acid,linolenic acid,oleic acid,cis-Zeatin riboside,3-Pgsgd,momordicoside F2,Momordicoside I,caffeic acid,5-Hydroxytryptamine,goyasaponin I,goyasaponin II,myristic acid,(3R,6E)-nerolidol,sinapinic acid,goyaglycoside c,goyaglycoside d,momordicoside F1,Momordicoside G,Momordicoside	AR,HMGCR,CYP51A1,CHRM2,CYP2C19,NR1H3,SLC6A2,CYP17A1,ESR1,SLC6A4,SREBF2,PTPN1,NPC1L1,RORC,CYP19A1,ACHE,BCHE,ESR2,NR1I3,SHBG,CES2,SQLE,SERPINA6,RORA,PTPN6,DHCR7,PTPN2,PREP,POLB,FNTAFNTB,NR1H2,FABP4,PPARG,PPARA,FABP3,FABP5,PPARD,FABP1,HSD11B1,G6PD,PTGES,VDR,PGR,ADORA3,MAPK3,PTPN11,AKR1B10,GLRA1,PDE4D,PTPRF,UGT2B7,PLA2G1B,ACP1,NOS2,HSD11B2,TOP2A,PTGER1,PTGER2,CDC25A,BACE1,ATP12A,CDC25B,SCD,CNR1,NR3C1,PRKCH,IDO1,PER2,PSEN2PSENENNCSTNAPH1APSEN1APH1B,HSD17B2,GRM5,SLC10A2,GCGR,PDE2A,PDE10A,MAPK14,NPY5R,TTL,AKR1C3,GRM2,AVPR1A,SLC6A3,MAPK8,KCNA5,KCNA3,OPRL1,PRCP,TRPV1,FDFT1,SCN9A,ABHD6,EPHX2,KCNH2,JAK3,PTGS2,IL6ST,CYP2C9,CYP3A4,TNK2,PABPC1,F2R,PGGT1B,HRH3,KDR,HIF1A,CCR9,OXTR,CYP11B1,CCR1,CYP11B2,HSD17B3,TRPA1,CHRM1,CHRM3,BRS3,CASR,MAP3K11,MDM2,SIGMAR1,PARP1,GCK,FKBP1A,GABBR2GABBR1,PRKCD,NPY2R,EIF2AK1,LRRK2,ABCC9,PGGT1BFNTA,LIPE,AVPR2,P2RX7,EGFR,HTR2A,PKM,LYPLA2,PYGL,AKR1B1,ADORA1,C5AR1,CHRNA7,ADORA2A,OPRM1,OPRD1,OPRK1,APP,MET,GLUL,ICMT,LYPLA1,HTR6,S1PR3,PIK3CB,PIK3CA,RAC1,FYN,LCK,KDM4E,CPA1,THRA,THRB,CA2,FFAR1,CPA3,CYP1A2,TH,TYR,MIF,PTPRA,KDM3A,KDM6B,FTO,KDM4A,KDM4C,BCL2L1,STAT3,IL2,F2,GLI1,JUN,PPM1B,PPP1CC,PPP2CA,PPP2R5A,ATP1A1,PRKCB,PRKCE,PRKCQ,HTR2B,HTR4,HTR1B,HTR1D,DRD1,HTR1A,HTR1E,HTR2C,DRD3,HTR7,HTR5A,HTR3A,MTNR1A,MTNR1B,ADRA2A,ADRA2C,FLT4,IGF1R,ADRB1,HRH1,LTA4H,DRD2,MPO,ANPEP,HRH2,FAAH,ADRA2B,QDPR,PNMT,ADRA1B,NR3C2,CHRNA3CHRNB4,AOC3,MAOB,KAT2B,TRPM8,MAOA,PLA2G2A,AADAT,KYAT3,NQO2,DPP7,CHRNA4,ADRA1A,NOX4,CDK5R1CDK5,XDH,FLT3,CCNB3CDK1CCNB1CCNB2,CDK6,SYK,GSK3B,ABCC1,HSD17B1,TTR,CSNK2A1,CFTR,CYP1B1,ABCG2,TNKS2,TNKS,ALOX5,CA7,CA12,ABCB1,ALOX12,CA4,PTPRS,GLO1,MMP9,MMP2,MMP12,CD38,TOP1,ARG1,ESRRA,PFKFB3,AMY1A,GRK6,ALOX15,AHR,CA1,CA9,CBR1,TERT,PIM1,CDK1,AURKB,TBXAS1,NAE1,PLK1,ALK,AXL,CDK2,GPR35,DAPK1,MPG,SLC22A12,ST6GAL1,PLG,DRD4,PIK3R1,SRC,PTK2,MMP13,MMP3,CA3,CA6,PKN1,CA14,NEK2,CXCR1,CAMK2B,AKT1,NEK6,ALDH2,MGAM,ESRRB,PTGS1,TLR9,PON1,CA5B,STS,HSP90AB1,CDC7,DUSP3,MMP1,CA5A,HCAR2,CA13,TLR4,ERBB2,MAPK1,AKR1C4,AKR1C2,ELANE,F3,NFE2L2,CHEK1,MTOR,MAP2K1,GBA,BCL2A1,CSF1R,AURKA,GRB2,ADK,PRF1,PDPK1,SLC5A2,HDAC6,HDAC1,HDAC4,JAK2,RASGRP3,AGTR1,CTSL,CTSB,GLRA2,CD274,SLC33A1,PRKCA,PTGFR,PTGIR,PTGER3,SLC22A6,PTGER4,CFD,ITGAL,PTGDR,MAP3K14,JAK1,DNTT,PDE4B,SMO,CDK2CCNA1CCNA2,NAMPT,TACR1,MAPK10,CDK4,CCR2,MAPK9,CCND1CDK4,CDK1CCNB1,LIMK2,METAP1,SLC29A1,ADORA2B,SLC5A1,HSPA8,HSPA5,DPP4,GAPDH,HK2,HK1,MMP7,MMP8,ADA,SETD7,AMD1,OGA,DNPH1,SRM,LGALS3,LGALS7,LGALS9,ADAM17,HSP90AA1,HRAS,MME,SETD2,CARM1,PRMT1,CTSK,CTSS,IGFBP3,SLC5A4,SLC28A2,SHH,DGAT1,NR1H4,PCSK7,HPGDS,PDGFRB,KIT,INSR,PLK4,TEK,MAP3K8,BRAF,EPHB4,HSPA1A,NUAK1,FGR,LYN,GSR,TNNC1TNNT2TNNI3,DAO,SNCA,CYP1A1,TUBB1,RELA,SLC16A1,TUBB3,FBP1,AMPD3,CD81,LTB4R,SRD5A2,CYP26B1,CYP26A1,PTGDR2,PTGES2,CES1,USP1,FUT7,LDHA,LDHB,SERPINE1,COMT,TPMT,ST3GAL3,FUT4,BCL2,MMP14,MAPT,DNMT1,DYRK1A,PGD,STAT1,GABRA1GABRB2GABRG2,KLK1,KLK2,TAS2R31,PGF,VEGF

	L,momordicine II,palmitoleic acid,palmitic acid,pentadecylic acid,t-cinnamic acid,tridecanoic acid,charantagenin D,goyaglycoside a,goyaglycoside b,Momordicoside K,lauric acid,ferulic acid,L-methionine	A,DNM1,ALB,ERN1,TACR2,CNR2,PTAFR,GC,REN,CYP24A1,CYP27B1,CTSD,CTSE,PGA5,FGFR1,GRIA1,KIF11,CCKBR,ROS1,INCENPAURKB,TTK,BRD4,BRD3,CHRM4,CHRM5,EZH2,PDE9A,PDE1C,SORD,PDK1,P2RX3,CXCR3,GPBAR1,WEE1,TYRO3,ITK,S1PR1,ABL1,PDGFRAPDGFRB,MAPK11,AGTR2,PIK3CAPIK3R1,HDAC8,SLC8A1,PDE5A,PLA2G2C,PIK3C2A,PIK3C3,MAP3K9,PIK3CD,PIK3CG,ALOX5AP,PIK3C2B,TYMS,PDE6A,RPS6KB1,MCHR1,HSD3B2,FASN,GNRHR,FLT1,HCRTR2,HCRTR1,CRHR1,PIM2,TACR3,GABRA5,CCNT1,PDE3A,PDE3B,CACNA2D1,CACNA2D2,RASGRP1,GPR55,GPR18,PDE7A,RET,F10,MST1R,FGFR3,ZAP70,NTRK1,TYK2,GYS1,NR1I2,ADAMTS5,ADAMTS4,PPM1A,PTPA,AKR1C1,AKR1A1,GABRB3GABRA3GABRG2,GABRB3GABRG2GABRA1,GABRB3GABRG2GABRA5,GABRA2GABRB3GABRG2,MERTK,SCARB1,MDM4,PRKCG,BMX,FGFR4,NTRK2,TIE1,TBXA2R,GSK3A,CCNE2CDK2CCNE1,FGFR2,CAMK2D,CCNCCDK8,CDK8,COL4A3BP,PYGM,IDH1,PTK6,CHUK,IRAK4,TNF,SRD5A1,MPEG1,MAP3K10,UGCG,SLC6A1,ITGALICAM1ITGB2,SLC6A9,FABP2,PHF8,GABBR1,KDM5C,KDM2A,GSTK1,HAO1,FFAR4,GABRA2GABRB2GABRG2,CDC45,RXRA,IMPDH2,EDNRA,PTPRC,RBP4,RARA,RARG,RARB,CMA1,CTSG,CXCL8,SAE1UBA2,MCL1,BMP1,NOS1,KYNU,KMO,NOS3,EPHX1,IL6,ENPP2,RXRB,PLA2G4A,RORB,HNF4A,DAGLA,OXER1,PRKAG1PRKAB1PRKAA2,RNPEP,OAT,PEPD,BHMT2,SF3B3,ACVRL1,ROCK2,CALCRL,MC4R,ACACA,ACACB,NTRK3,UPP1,ALPL,CASP3,CASP7,PAK1,ERBB4,CDK7,CDK9,ABCB11,F7,PI4KB,SIRT2,IKBKE,MKNK2,TBK1,MAP2,ADRB2,ADRB3,GRM1,CAPN2,CAPN1,ITGB7ITGA4,CCNE1CDK2,CDK5,LIMK1,ROCK1,PLK3,KLK3,CCNA2CDK2,P2RY12,RPS6KA3,SOAT1,RPS6KA4,CSNK1E,NLK,CDK9CCNT1,CSNK1A1,LSS,CCND3CCND1CDK4CCND2,CYP2D6,IMPDH1,EIF4A1,DHFR,MYLK,APEX1,RXRG,KEAP1,PLA2G5,PLA2G10,HSD17B14,CLK1,DYRK1B,ADCY5,YWHAG,VCP,PSEN1,PDE4A,NR0B2,FOLH1,EGLN1,CTNNB1,ACLY,SLC13A5,ILK,CAMKK2,ACE,ECE1,PIN1,HDAC2,HDAC10,ARAF,RAF1,CSK,TNIK,RIPK2,PDGFRA,AURKC,RIPK1,YES1,HCK,CLK2,HIPK4,CAMK1,STK16,DYRK2,HIPK2,HIPK3,MYLK4,HIPK1,EPHB6,RIOK2,OXSR1,BLK,CSNK1G2,MAP2K4,MAP2K2,MAP3K20,ABL2,SLK,FRK,TGFBR2,STK36,MAP4K5,MAP2K5,DDR2,DDR1,EIF2AK4,FES,ACVR2B,BMPR1B,MINK1,TESK1,NEK11,DCLK1,SIK2,SRMS,CDK19,SIK1,DCLK3,SGK3,CCKAR,PCK1,PTK2B,BDKRB1,MGLL,TAOK2,CDKL3,EPHA2,MYLK2,MAPK13,EPHB2,STK10,EPHA5,EPHA4,EPHA8,CDK3,EPHA6,EPHA7,MYLK3,MAPK12,MKNK1,RPS6KA6,EPHA3,EPHB1,MAPK15,TNNI3K,TAOK1,TNK1,CIT

SALVIAE MILTIORRHIZAE RADIX ET RHIZOMA	1,2,5,6-tetrahydrotanshinone,Poriferasterol,poriferast-5-en-3beta-ol,isoimperatorin,sugiol,Dehydrotanshinone II A,Baicalin,digallate,luteolin,α-amyrin,5,6-dihydroxy-7-isopropyl-1,1-dimethyl-2,3-dihydrophenanthren-4-one,2-isopropyl-8-methylphenanthrene-3,4-dione,3α-hydroxytanshinone Ⅱ a,(E)-3-[2-(3,4-dihydroxyphenyl)-7-hydroxy-benzofuran-4-yl]acrylic acid,4-methylenemiltirone,2-(4-hydroxy-3-methoxyphenyl)-5-(3-hydroxypropyl)-7-methoxy-3-benzofurancarboxaldehyde,6-o-syringyl-8-o-acetyl shanzhiside methyl ester,formyltanshinone,3-beta-Hydroxymethyllenetanshiquinone,Methylenetanshinquinone,przewalskin a,przewalskin b,Przewaquinone B,przewaquinone c,(6S,7R)-6,7-dihydroxy-1,6-dimethyl-8,9-dihydro-7H-naphtho[8,7-g]benzofuran-10,11-dione,przewaquinone f,sclareol,tanshinaldehyde,Danshenol B,Danshenol A,Salvilenone,cryptotanshinone,dan-shexinkum d,danshenspiroketallactone,deoxyneocryptotanshinone,dihydrotanshinlactone,dihydrotanshinone Ⅰ,epidanshenspiroketallactone,C09092,isocryptotanshi-none,Isotanshinone II,manool,microstegiol,miltionone Ⅰ,miltionone	HTR1A,HTR1B,HTR2A,HTR2C,HTR3A,MMP2,ACHE,AHSA1,ADCY2,GUSBP1,ADRA1A,ADRA1B,ADRA1D,ADRA2A,ADRA2B,ADRA2C,APP,AR,BCL2,BIRC4,BIRC5,BCL2L1,ADRB2,blaSHV,CALCR,KCNMA1,CALM,CA2,CASP3,CASP7,CASP9,CD40LG,CDK2,CDK4,TP53,PDEA,F7,F10,CCNA2,CDKN1A,CYP1A1,CYP1A2,CYP3A4,DRD5,DRD2,OPRD1,DPP4,TOP1,TOP2A,TOP2,DRD1,MDM2,EDN1,EDNRA,ECE1,EGFR,ESR1,ESR2,FASN,CCND1,CCNB1,GABRA1,GABRE,GABRG3,GABRA2,GABRA5,GABRA3,GABRA6,NR3C1,GSTP1,GSK3B,HSP90,HMOX1,MET,IGHG1,MCL1,INSR,ITGB3,ICAM1,IFNG,IL10,IL2,IL4,IL6,MMP1,NUF2,MMP9,NR3C2,MAPK1,MAPK14,PRKACA,PTPN1,CHRM1,CHRM2,CHRM3,CHRM4,CHRM5,OPRM1,MYC,CHRNA7,CHRNA2,NFKBIA,NOS2,NOS3,NCOA1,NCOA2,NR1I2,NPM1,PPARG,PIK3CG,PARP4,KCNH2,PGR,PCNA,PTGES,PTGS1,PTGS2,FOS,PIM1,AKT1,ERBB2,RB1,RXRA,CHEK1,STAT3,SCN5A,SLC6A3,SLC6A2,SLC6A4,SLC2A4,F2,TFAP4,RELA,PRSS1,TNF,TYR,VEGFA,XDH

	Ⅱ,miltipolone,Miltirone,miltirone Ⅱ,neocryptotanshinone ii,neocryptotanshinone,1-methyl-8,9-dihydro-7H-naphtho[5,6-g]benzofuran-6,10,11-trione,prolithospermic acid,(2R)-3-(3,4-dihydroxyphenyl)-2-[(Z)-3-(3,4-dihydroxyphenyl)acryloyl]oxy-propionic acid,(Z)-3-[2-[(E)-2-(3,4-dihydroxyphenyl)vinyl]-3,4-dihydroxy-phenyl]acrylic acid,salvianolic acid g,salvianolic acid j,salvilenone Ⅰ,salviolone,NSC 122421,(6S)-6-hydroxy-1-methyl-6-methylol-8,9-dihydro-7H-naphtho[8,7-g]benzofuran-10,11-quinone,Tanshindiol B,Przewaquinone E,tanshinone iia,(6S)-6-(hydroxymethyl)-1,6-dimethyl-8,9-dihydro-7H-naphtho[8,7-g]benzofuran-10,11-dione,tanshinone Ⅵ	

PUERARIAE LOBATAE RADIX	formononetin,beta-sitosterol,3'-Methoxydaidzein,Daidzein-4,7-diglucoside	ABCB1,ABCC1,ABCG2,ABL1,ACHE,ACP1,ADORA1,ADORA2A,ADORA3,ADRB2,AKR1B1,AKR1B10,ALDH2,ALOX12,ALOX15,ALOX5,AR,ATP12A,AURKB,AVPR1A,BACE1,BAD,BCHE,BCL2,BIRC5,BRAF,CA1,CA12,CA13,CA14,CA2,CA3,CA4,CA5A,CA5B,CA6,CA7,CA9,CBR1,CCNB3CDK1CCNB1CCNB2,CCR1,CCR4,CDC25B,CDC7,CES2,CHEK1,CHEK2,CHRM2,CNOT7,CRHR1,CXCR2,CXCR3,CYP17A1,CYP19A1,CYP1B1,CYP2C19,CYP51A1,DGAT1,DHCR7,DHCR7EBP,DHODH,DNM1,DRD2,DUSP3,EGFR,EPHB4,ERCC5,ESR1,ESR2,ESRRA,ESRRB,EZR,F10,F2,FABP1,FABP3,FABP4,FABP5,FDFT1,FEN1,FLT3,G6PD,GC,GCGR,GLRA1,GPR84,GRK6,GSK3B,HCK,HCRTR1,HCRTR2,HIF1A,HMGCR,HSD11B1,HSD11B2,HSD17B1,HSD17B2,HSP90AA1,HSP90AB1,HSP90B1,HTR2A,HTR2C,ICMT,IDO1,IGFBP1,IGFBP2,IGFBP3,IGFBP4,IGFBP5,IGFBP6,IKBKE,IL2,ITGALICAM1ITGB2,KCNMA1,KDM1A,KDM4A,KDM4C,KDM4D,KDM5C,KIF11,KIT,KLKB1,LIG1,LRRK2,MAOA,MAOB,MAPK3,MAPK8,MAST3,MCL1,MDM2,METAP1,MGAM,MGLL,MIF,NOS2,NOX4,NPC1L1,NR1H2,NR1H3,NR1I3,NR3C1,NR3C2,NTRK1,OPRD1,PDE4A,PDE4B,PDE4C,PFKFB3,PI4KB,PIM2,PIM3,PLA2G1B,PLA2G2A,PLA2G4A,PLAT,PLAU,PLG,POLB,PON1,PPARA,PPARD,PPARG,PREP,PRKCH,PRMT1,PRSS1,PTGDR,PTGER1,PTGER2,PTGER3,PTGER4,PTGES,PTGFR,PTGIR,PTGS1,PTK6,PTP4A3,PTPN1,PTPN11,PTPN2,PTPN6,PTPRF,PTPRS,PYGL,RAF1,RORA,RORC,RPS6KA3,S1PR1,S1PR3,SERPINA6,SHBG,SHH,SIGMAR1,SLC6A2,SLC6A3,SLC6A4,SMO,SNCA,SQLE,SRD5A2,SREBF2,STS,TBXA2R,TBXAS1,TERT,TLR8,TLR9,TNF,TNKS,TNKS2,TNNC1TNNT2TNNI3,TOP1,TOP2A,TYK2,TYR,UGT2B7,VDR,WEE1,XDH,ADRA1B,ADRA1D,AMH,ATP5F1B,BIRC2,CASP3,CASP8,CASP9,CCNA2,CDC25A,CDK2,CES1,CHRM1,CHRM3,CHRM4,CHRNA2,CHRNA7,COX2,CRYAB,CYP1A1,CYP27B1,DHCR24,DPP4,DRD1,DRD5,GABRA1,GABRA2,GABRA3,GABRA5,JUN,KCNH2,MAP2,MAPK14,NCOA1,NCOA2,ND6,NOS3,OPRM1,OSBP2,PDE3A,PGR,PIK3CG,PKIA,PRKACA,RXRA,SCN5A,SORD,TOP2B,UGT1A10,UGT1A7,UGT1A8,UGT1A9,ADRA1A,AOC2,BAX,CALM,CYP101A1,HSD3B1,HSD3B2,HSP90,IL4,MT-ND6,PDEA,PIM1,PRKCA,PTGS2,SIRT1,TFAP4,TGFB1,TOP2

POLYGONI MULTIFLORI RADIX PRAEPARATA	4-Methylcatechol,2,3,5,4'-Tetrahydroxystilbene 2-O-beta-D-glucoside,Isoorientin,Kaempferol,Centaurein,Quercetin,Emodin-8-glucoside,1,4-dihydroxy-2-methoxy-3-methyl-5-[(2S,3R,4S,5S,6R)-3,4,5-trihydroxy-6-(hydroxymethyl)oxan-2-yl]oxyanthracene-9,10-dione,Physcion,Emodin,Aloe emodin,Anhydroicaritin,Beta-sitosterol,Rhein	ACHE,CA2,CA12,CA3,DAO,ESR1,ESR2,HSD17B2,HSD17B1,PTPN22,HDAC6,AR,AKR1B1,CA7,NOX4,XDH,TYR,FLT3,ALOX5,ABCC1,AHR,ESRRA,ABCB1,CYP1B1,ABCG2,ADORA1,CA4,MAOA,GLO1,SYK,GSK3B,MMP9,MMP2,ALOX15,ALOX12,PTPRS,ADORA2A,CDK5R1CDK5,CCNB3CDK1CCNB1CCNB2,ARG1,GPR35,DAPK1,MPG,SLC22A12,TTR,AKR1B10,TNKS2,TNKS,CDK6,CDK2,CYP19A1,CSNK2A1,EGFR,AVPR2,IGF1R,F2,PIM1,AURKB,DRD4,MPO,PIK3R1,PYGL,CA1,SRC,PTK2,KDR,MMP13,MMP3,PLK1,CA6,CDK1,PKN1,CA14,CA9,MET,NEK2,CXCR1,CAMK2B,ALK,AKT1,NEK6,PLA2G1B,CA5A,BACE1,AXL,NUAK1,AKR1C2,AKR1C1,AKR1C3,AKR1C4,CA13,AKR1A1,APP,PARP1,MMP12,CD38,TOP1,PTGS2,CFTR,PFKFB3,AMY1A,GRK6,TERT,MAPT,TNF,IL2,ADRA2C,NMUR2,ADRA2A,RPS6KA3,NQO2,HSP90AA1,SLC28A2,IMPDH1,IMPDH2,ADORA2B,PLG,PDE5A,KDM4E,TOP2A,INSR,MYLK,PIK3CG,APEX1,EPHX2,TNNC1TNNT2TNNI3,SLC5A4,SLC5A2,ELANE,SLC29A1,SLC28A3,PTPN1,SLC5A1,ADORA3,LGALS3,LGALS9,MMP1,MMP7,MMP8,ADK,LGALS4,LGALS8,IGFBP3,LIMK1,PTP4A3,LCK,MCL1,DUSP3,BCL2,FTO,EIF2AK2,MME,NQO1,CHRNA7,HDAC8,HDAC1,MMP10,KDM1A,ADAM17,MAPK8,MMP16,MMP14,KCNMA1,CTSV,BAD,MAP2K1,BCHE,PLA2G7,GUSB,NOTUM,LRRK2,HDAC5,HDAC7,HDAC4,HDAC9,ADAMTS5,ADAMTS4,PDE4B,PDE4C,ANPEP,PIK3CD,CCNE1CDK2,ERBB2,FLT1,PDGFRB,FLT4,PDGFRA,FNTAFNTB,FASN,CRHR1,DRD3,CBR1,TBXAS1,MGAM,HTR2C,ESRRB,LDHA,LDHB,PPARG,HSP90,PRKACA,DPP4,PRSS1,NOS3,GABRA2,TOP2,CALM,TFAP4,KCNH2,TGFB1,blaSHV,ADH1C,NFE2L2,NOS2,PTGS1,NCOA2,PGR,CHRM1,SLC6A2,CHRM2,ADRA1B,GABRA1,F7,RELA,IKBKB,BAX,AHSA1,CASP3,STAT1,HMOX1,CYP3A4,CYP1A2,CYP1A1,ICAM1,SELE,VCAM1,NR1I2,HAS2,GSTP1,PSMD3,SLC2A4,NR1I3,DIO1,GSTM1,GSTM2,SLPI,SCN5A,ADRB2,RXRA,CCND1,BCL2L1,CDKN1A,EIF6,CASP9,PLAU,MAPK1,IL10,EGF,RB1,IL6,TP53,ELK1,NFKBIA,POR,ODC1,CASP8,RAF1,PRKCA,HIF1A,RUNX1T1,ACACA,CAV1,MYC,F3,GJA1,IL1B,CCL2,PTGER3,CXCL8,PRKCB,BIRC5,DUOX2,HSPB1,CCNB1,PLAT,THBD,SERPINE1,IFNG,IL1A,NCF1,CXCL11,CXCL2,DCAF5,CHEK2,CLDN4,PPARA,PPARD,HSF1,CRP,CXCL10,CHUK,SPP1,RUNX2,RASSF1,E2F1,E2F2,ACP3,CTSD,CD40LG,IRF1,ERBB3,PON1,PCOLCE,NPEPPS,HK2,NKX31,RASA1,NCOA1,PKIA,PRKCE,PRKCD,CSF2,ACTA2,BTK,SLC2A1,PCNA,CHRM3,CHRM5,MAPK14,CHEK1,RXRB,CCNA2,LYZ,cobT,CDC2,Ppp3cb,PRXC1A,GUSBP1,F10,MAOB,COL1A1,PTEN,IGHG1,VEGFA,FOS,Cdkn2a,SOD1,HERC5,HSPA5,SULT1E1,COL3A1,gyrB,IGF2

Table S2. The information of the top 10 GO-BP pathways obtained through screening.

Term	Description	LogP	Symbols
GO:1901699	cellular response to nitrogen compound	-82.33256315	ABL1,ACHE,PARP1,ADRB2,GRK2,AHR,AKT1,APOA2,APP,BCL2L1,CA2,CA3,CACNA1S,CASP3,CASP7,CAV1,CCNA2,CDK2,CDK5,CFTR,CHRM1,CHRM2,CHRM3,CHRNA7,COMT,CRHR1,CTNNB1,DRD1,DRD2,DRD3,DRD4,DRD5,EDN1,EDNRA,FOS,MTOR,GJA1,GRB2,GSK3B,GSTM2,GSTP1,HDAC2,HRAS,HSF1,HTR1A,HTR1B,HTR2A,HTR2C,HTR3A,HTR7,ICAM1,IGF1R,IGF2,INSR,ITGB3,JAK1,JAK2,JAK3,MDM2,MMP3,ABCC1,NFE2L2,NFKB1,NPM1,OPRM1,PDE3A,PDPK1,ABCB1,PIK3CA,PIK3CG,PIK3R1,PLA2G1B,POR,PPARG,PRKACA,PRKCB,PRKCD,PRKDC,MAPK1,MAPK3,PTGER1,PTK2,PTPN1,PTPN2,RAF1,RB1,RELA,RPS6KB1,SLC2A4,SLC6A4,SOD1,SRC,STAT1,STAT3,SYK,TGFB1,TNF,TOP1,TOP2B,TP53,HSP90B1,VCAM1,CDK5R1,HDAC9,HDAC5,KDM1A,BACE1,PTPN22,TLR9,SLC22A12,LRRK2,ACACA,ADRA2A,AR,AVPR2,CASP9,CTSL,AKR1C1,ESR1,ESR2,ESRRA,ESRRB,FLT3,NR3C1,NR3C2,PGR,PIM1,PPARA,PPARD,PRKCE,PTGER2,RXRA,RXRB,RXRG,THRB,NR1H2,AKR1C3,NCOA1,HDAC6,NR1H3,NCOA2,SIRT1,ALPL,BMP7,CHUK,COL1A1,COL3A1,MAPK14,F7,FABP3,IL10,EIF6,LTA4H,PRKCA,ROCK1,SLC2A1
GO:0009410	response to xenobiotic stimulus	-73.31573732	ABL1,ADORA2A,ADRA1A,AHR,ALDH2,AMH,BCHE,CCND1,BCL2,BRAF,CA9,CASP3,CBR1,CCNB1,CD38,CDK4,CHUK,COL1A1,COMT,CTNNB1,CYP1A1,CYP1A2,CYP1B1,CYP2C19,CYP3A4,AKR1C1,NQO1,DRD1,DRD2,DRD3,E2F1,EDN1,SLC29A1,FABP3,FOS,GABRG3,GSTM1,GSTM2,GSTP1,HDAC2,HSF1,HSP90AA1,HTR1B,HTR2A,IL1B,IL10,ITGB3,JUN,KCNH2,LCK,MDM2,MMP2,MMP7,ABCC1,MYC,NFE2L2,NOS2,PCNA,PDE3A,PDE4A,PDE4B,ABCB1,POR,PRKCE,RB1,RELA,RORA,RORC,SLC6A2,SLC6A3,SLC6A4,SOD1,STAT1,ADAM17,TGFB1,TGFBR2,TNF,TOP1,TP53,UGT2B7,NR1I2,HDAC5,AGPAT2,CHEK2,UGT1A10,UGT1A8,UGT1A7,UGT1A9,SLC28A3,SLC22A12

GO:0071396	cellular response to lipid	-60.16256988	ABL1,ACACA,ADCY2,AHR,AKT1,AR,AXL,BAD,CASP1,CASP7,CASP9,CCNA2,CCNB1,CDK4,CES1,CFTR,COL1A1,MAPK14,CSF2,CYP27B1,AKR1C1,DRD2,E2F1,EDN1,EGFR,ELK1,ESR1,ESR2,ESRRA,ESRRB,FLT3,FOS,NR3C1,GSK3B,GSTP1,HDAC2,HSF1,IL1A,IL1B,IL6,CXCL8,IL10,CXCL10,JAK2,NR3C2,MMP2,MMP3,MMP8,MMP9,NFKB1,NFKBIA,NOS2,NOS3,SERPINE1,PDE3A,PDE4B,PGR,ABCB1,PIM1,PPARA,PPARD,PRKCD,PRKCE,MAPK1,MAPK3,MAPK8,PTGER2,RELA,RET,RORA,RORC,RPS6KB1,RXRA,RXRB,RXRG,CCL2,SLC6A4,SPP1,SRC,SYK,TGFB1,TNF,AKR1C3,NCOA1,HDAC5,NR1H3,PTPN22,TLR9,PIM3,NCF1,ALPL,BTK,CASP3,CASP8,CYP1A1,NQO1,ELANE,CXCL2,LGALS9,MPO,PTGER1,RPS6KA3,SELE,SLPI,ADAM17,THBD,VCAM1,NR1I2,MAPKAPK2,APP,BMP2,CA3,CAV1,CRP,DAO,F2,FABP4,MTOR,IGHG1,LGALS4,MMP7,PLA2G1B,PYGL,ADAMTS4,ADAMTS5,HSPA5,TP53
GO:0032870	cellular response to hormone stimulus	-58.70053336	ACACA,PARP1,ADRA2A,AKT1,AR,AVPR2,CA2,CA3,CASP9,CAV1,CCNA2,CRHR1,CTSL,AKR1C1,EDN1,EDNRA,ESR1,ESR2,ESRRA,ESRRB,FLT3,FOS,MTOR,GRB2,NR3C1,GSK3B,GSTP1,HDAC2,HRAS,HSF1,IGF1R,IGF2,INSR,ITGB3,JAK1,JAK2,JAK3,MDM2,NR3C2,NFE2L2,NFKB1,PDE3A,PDPK1,PGR,ABCB1,PIK3CA,PIM1,PIK3R1,PLA2G1B,POR,PPARA,PPARD,PPARG,PRKACA,PRKCD,PRKCE,PRKDC,MAPK1,MAPK3,PTGER2,PTK2,PTPN1,PTPN2,RAF1,RB1,RELA,RPS6KB1,RXRA,RXRB,RXRG,SLC2A4,SRC,STAT1,STAT3,TGFB1,THRB,TOP1,NR1H2,AKR1C3,NCOA1,HDAC9,HDAC6,HDAC5,NR1H3,NCOA2,SIRT1,SLC22A12
GO:2000147	positive regulation of cell motility	-58.53744708	ABL1,ACTA2,ADRA2A,AKT1,APP,ATP5F1B,BCL2,BMP2,BMP7,CASP8,CAV1,COL1A1,CLDN4,CSF2,DRD1,EDN1,EGF,EGFR,F3,F7,F10,FLT1,FLT4,MTOR,GSK3B,HAS2,HIF1A,HMOX1,HRAS,HSPA5,HSPB1,ICAM1,IFNG,IGF1R,IL1B,IL4,IL6,CXCL8,CXCL10,INSR,ITGB3,JAK2,JUN,KDR,LGALS3,LGALS9,MDM2,MET,MMP2,MMP7,MMP9,MMP14,MYLK,NFE2L2,NOS3,SERPINE1,PDGFRA,PDGFRB,PDPK1,PGF,PIK3CB,PIK3CD,PIK3CG,PIK3R1,PLAU,PLG,PRKCA,PRKCE,MAPK1,MAPK3,MAP2K1,MAP2K2,PTGS2,PTK2,RET,CCL2,SELE,SRC,STAT3,ADAM17,TERT,TGFB1,TGFBR2,TNF,VEGFA,PLA2G7,HDAC9,HDAC6,SIRT1,DUOX2,HDAC7,PARP1,ADRA2B,ADRA2C,ALK,AMH,AR,AXL,BRAF,CASP3,RUNX2,COL3A1,MAPK14,CTNNB1,EPHB4,ERBB2,ERBB3,FLT3,FNTA,FOS,GRB2,IGF2,JAK1,JAK3,LCK,PIK3CA,PLAT,PPARG,PTPN1,PTPN2,RAF1,RASA1,RELA,RPS6KB1,SYK,TP53,CDK5R1,MAPKAPK2,CCNA2,E2F1,ELK1,ERN1,NR3C1,GSTP1,HDAC1,HDAC2,IL10,MAPT,MYC,OPRD1,PDE3A,ROCK1,VCAM1,AHR,ANPEP,BAX,COMT,CYP1B1,EDNRA,RORA,SHH,TNNI3,ESR1,PGR,PRKACA

GO:0030335	positive regulation of cell migration	-58.07598808	ABL1,ACTA2,ADRA2A,AKT1,APP,ATP5F1B,BCL2,BMP2,BMP7,CASP8,CAV1,COL1A1,CLDN4,CSF2,DRD1,EDN1,EGF,EGFR,F3,F7,F10,FLT1,FLT4,MTOR,GSK3B,HAS2,HIF1A,HMOX1,HRAS,HSPA5,HSPB1,ICAM1,IFNG,IGF1R,IL1B,IL4,IL6,CXCL8,CXCL10,INSR,ITGB3,JAK2,JUN,KDR,LGALS3,LGALS9,MDM2,MET,MMP2,MMP7,MMP9,MMP14,MYLK,NFE2L2,NOS3,SERPINE1,PDGFRA,PDGFRB,PDPK1,PGF,PIK3CB,PIK3CD,PIK3CG,PIK3R1,PLAU,PLG,PRKCA,PRKCE,MAPK1,MAPK3,MAP2K1,PTGS2,PTK2,RET,CCL2,SELE,SRC,STAT3,ADAM17,TERT,TGFB1,TGFBR2,TNF,VEGFA,PLA2G7,HDAC9,HDAC6,SIRT1,HDAC7
GO:0040017	positive regulation of locomotion	-57.5079996	ABL1,ACTA2,ADRA2A,AKT1,APP,ATP5F1B,BCL2,BMP2,BMP7,CASP8,CAV1,COL1A1,CLDN4,CSF2,DRD1,EDN1,EGF,EGFR,F3,F7,F10,FLT1,FLT4,MTOR,GSK3B,HAS2,HIF1A,HMOX1,HRAS,HSPA5,HSPB1,ICAM1,IFNG,IGF1R,IL1B,IL4,IL6,CXCL8,CXCL10,INSR,ITGB3,JAK2,JUN,KDR,LGALS3,LGALS9,MDM2,MET,MMP2,MMP7,MMP9,MMP14,MYLK,NFE2L2,NOS3,SERPINE1,PDGFRA,PDGFRB,PDPK1,PGF,PIK3CB,PIK3CD,PIK3CG,PIK3R1,PLAU,PLG,PRKCA,PRKCE,MAPK1,MAPK3,MAP2K1,MAP2K2,PTGS2,PTK2,RET,CCL2,SELE,SRC,STAT3,ADAM17,TERT,TGFB1,TGFBR2,TNF,VEGFA,PLA2G7,HDAC9,HDAC6,SIRT1,DUOX2,HDAC7
GO:0043408	regulation of MAPK cascade	-54.32015371	ABL1,ACTA2,ADORA1,ADORA2A,ADRA1D,ADRA1B,ADRA1A,ADRA2A,ADRA2B,ADRA2C,ADRB1,ADRB2,ALOX15,APP,AR,BMP2,BRAF,CALCR,CAV1,CHRNA7,CTNNB1,DRD1,DRD2,DRD4,DRD5,EDN1,EGF,EGFR,ELANE,ERBB2,ERBB3,ERN1,FLT1,FLT3,FLT4,GRB2,GRM4,GSTP1,HMGCR,HRAS,HTR2A,HTR2C,ICAM1,IGF1R,IGF2,IGFBP3,IL1A,IL1B,IL6,INSR,ITGB3,JAK2,JUN,KDR,LCK,LGALS9,MIF,MYC,OPRM1,PDGFRA,PDGFRB,PIK3CB,PIK3CG,PLA2G1B,PPARG,PRKCA,PRKCD,PRKCE,MAPK1,MAPK3,MAP2K1,MAP2K2,EIF2AK2,PTPN1,PTPN2,PTPN6,RAF1,RET,ROCK1,SOD1,SRC,SYK,TGFB1,TNF,VEGFA,PDE8B,PTPN22,NOX4,TLR9,LRRK2
GO:0043410	positive regulation of MAPK cascade	-53.3642029	ABL1,ACTA2,ADORA1,ADORA2A,ADRA1D,ADRA1B,ADRA1A,ADRA2A,ADRA2B,ADRA2C,ADRB1,ADRB2,ALOX15,APP,AR,BMP2,BRAF,CALCR,CHRNA7,CTNNB1,DRD1,DRD2,DRD4,DRD5,EDN1,EGF,EGFR,ELANE,ERBB2,ERBB3,ERN1,FLT1,FLT3,FLT4,GRM4,HRAS,HTR2A,HTR2C,ICAM1,IGF1R,IGF2,IGFBP3,IL1B,IL6,INSR,ITGB3,JAK2,JUN,KDR,LCK,LGALS9,MIF,OPRM1,PDGFRA,PDGFRB,PIK3CG,PLA2G1B,PRKCA,PRKCE,MAPK3,MAP2K1,EIF2AK2,PTPN1,RAF1,RET,ROCK1,SOD1,SRC,SYK,TGFB1,TNF,VEGFA,PDE8B,PTPN22,NOX4,TLR9,LRRK2

GO:0070482	response to oxygen levels	-52.92370106	ADORA1,AKT1,BIRC2,BAD,BCL2,BMP2,BMP7,CA3,CA9,CASP3,CASP9,CAV1,CCNA2,CCNB1,CD38,CHRNA7,COL1A1,COMT,CRYAB,CYP1A1,DPP4,DRD2,E2F1,EDN1,EDNRA,SLC29A1,F7,FOS,MTOR,HDAC2,HIF1A,HK2,HSF1,KCNMA1,MDM2,MMP2,MMP14,COX2,MYC,NFE2L2,NOS2,OPRD1,PGF,ABCB1,PLAT,PLAU,POLB,PPARA,PPARD,PPARG,PRKCE,PTGS2,PTPN1,RORA,SLC2A1,SLC2A4,SLC6A4,STAT3,ADAM17,TERT,TGFB1,TNF,TP53,HSP90B1,VCAM1,VEGFA,PARP2,SIRT1

Table S3. The information of the top 10 GO-CC pathways obtained through screening.

Term	Description	LogP	Symbols
GO:0045121	membrane raft	-28.95636789	ADCY2,ADRA1B,ADRA1A,APP,BTK,CAV1,CHRNA7,CNR1,CTNNB1,CTSD,DPP4,EGFR,GJA1,HAS2,HTR2A,ICAM1,IKBKB,INSR,JAK2,KCNMA1,KDR,LCK,LDHB,MAPT,MME,NOS3,PRKACA,MAPK1,MAPK3,SCN5A,SELE,SHH,SLC2A1,SLC2A4,SLC6A3,SLC6A4,SRC,ADAM17,TGFBR2,TNF,ABCG2,HDAC6,BACE1,LRRK2
GO:0098857	membrane microdomain	-28.8272819	ADCY2,ADRA1B,ADRA1A,APP,BTK,CAV1,CHRNA7,CNR1,CTNNB1,CTSD,DPP4,EGFR,GJA1,HAS2,HTR2A,ICAM1,IKBKB,INSR,JAK2,KCNMA1,KDR,LCK,LDHB,MAPT,MME,NOS3,PRKACA,MAPK1,MAPK3,SCN5A,SELE,SHH,SLC2A1,SLC2A4,SLC6A3,SLC6A4,SRC,ADAM17,TGFBR2,TNF,ABCG2,HDAC6,BACE1,LRRK2
GO:0043235	receptor complex	-22.68888635	ADRA2A,ADRB2,AHR,ALK,BIRC2,APP,AXL,BMP2,CALCR,CHRNA7,CHUK,CSF2,DRD1,DRD2,EGFR,EPHB4,ERBB2,ERBB3,FLT1,FLT3,FLT4,GABRA1,GABRA2,GABRA3,GABRA5,GABRG3,HSP90AB1,HTR1B,HTR2A,HTR2C,HTR3A,IGF1R,IKBKB,IL6,INSR,ITGB3,JAK1,JAK2,KDR,MET,NR3C2,PDGFRA,PDGFRB,PPARG,PTPN6,RET,RXRA,SYK,TGFBR2,NR1H3,CACNA1S,CASP3,CASP8,CAV1,CTNNB1,GJA1,GRB2,KCNH2,KCNMA1,PDE4B,PRKCA,SCN5A,SLC6A3,VCAM1,DUOX2,NOX4,NCF1
GO:0030425	dendrite	-21.58552993	ABL1,ADCY2,ADORA1,ADORA2A,ADORA3,APP,CDK5,CHRM1,CHRM2,CHRM3,CHRNA7,COMT,CRYAB,DRD1,DRD2,DRD4,DYRK1A,ELK1,MTOR,GABRA1,GABRA2,GABRA3,GABRA5,GABRG3,GLRA1,GSK3B,HSP90AA1,HSP90AB1,HTR1A,HTR1B,HTR2A,HTR2C,HTR7,INSR,MAP2,MAPT,MME,OPRD1,OPRM1,PDE4B,MAPK8,MAP2K1,PTEN,SOD1,STAT1,CDK5R1,HDAC6,TUBB3,BACE1,OSBP2,LRRK2,NCF1,ADRA2A,CALCR,CNR1,CYP17A1,HIF1A,HSPB1,IGF1R,MYC,PRKCB,PTPRS,RET,SLC6A2,SLC6A3,TGFB1,SIGMAR1,ACTA2,BRAF,CASP3,CASP8,HDAC1,TNF,TOP1
GO:0097447	dendritic tree	-21.52296253	ABL1,ADCY2,ADORA1,ADORA2A,ADORA3,APP,CDK5,CHRM1,CHRM2,CHRM3,CHRNA7,COMT,CRYAB,DRD1,DRD2,DRD4,DYRK1A,ELK1,MTOR,GABRA1,GABRA2,GABRA3,GABRA5,GABRG3,GLRA1,GSK3B,HSP90AA1,HSP90AB1,HTR1A,HTR1B,HTR2A,HTR2C,HTR7,INSR,MAP2,MAPT,MME,OPRD1,OPRM1,PDE4B,MAPK8,MAP2K1,PTEN,SOD1,STAT1,CDK5R1,HDAC6,TUBB3,BACE1,OSBP2,LRRK2,NCF1

GO:1902911	protein kinase complex	-20.42297113	CCND1,CCNA2,CCNB1,CCND2,CCND3,CCNE1,CDK1,CDK2,CDK4,CDK5,CDK6,CDKN1A,CHUK,CSNK2A1,DAPK1,ERN1,MTOR,IGF1R,IKBKB,INSR,PCNA,PRKACA,PRKDC,RB1,TGFBR2,CDK5R1,CCNA1,CCNB2,PFKFB3,PIK3CA,PIK3CB,PIK3CD,PIK3CG,PIK3R1,TERT
GO:1902554	serine/threonine protein kinase complex	-19.20955851	CCND1,CCNA2,CCNB1,CCND2,CCND3,CCNE1,CDK1,CDK2,CDK4,CDK5,CDK6,CDKN1A,CHUK,CSNK2A1,DAPK1,ERN1,MTOR,IKBKB,PCNA,PRKACA,PRKDC,RB1,TGFBR2,CDK5R1,CCNA1,CCNB2
GO:0061695	transferase complex, transferring phosphorus-containing groups	-18.70742605	CCND1,CCNA2,CCNB1,CCND2,CCND3,CCNE1,CDK1,CDK2,CDK4,CDK5,CDK6,CDKN1A,CHUK,CSNK2A1,DAPK1,ERN1,MTOR,IGF1R,IKBKB,INSR,PCNA,PFKFB3,PIK3CA,PIK3CB,PIK3CD,PIK3CG,PIK3R1,PRKACA,PRKDC,RB1,TERT,TGFBR2,CDK5R1,CCNA1,CCNB2
GO:0098794	postsynapse	-18.11169372	ABL1,ADORA1,ADORA2A,ADRA2A,GRK2,AKT1,APP,BRAF,CAMK2B,CASP3,CDK5,CHRM1,CHRM2,CHRM3,CHRNA7,CRHR1,CRYAB,CTNNB1,DAPK1,DRD1,DRD2,DRD4,SLC29A1,GABRA1,GABRA2,GABRA3,GABRA5,GABRG3,GLRA1,GSK3B,HTR2A,HTR3A,ITGB3,JAK2,KCNMA1,LIMK1,MAPT,MDM2,MET,OPRD1,PDE4B,PDPK1,PRKACA,MAP2K1,PTEN,PTPN1,PTPRS,SLC6A3,SLC6A4,STAT3,CDK5R1,SIGMAR1,LRRK2,ADORA3,CNR1,ERBB2,HTR1B,SLC6A2,ADORA2B,BCL2L1,DAO,ELK1,GRM4,MME,PRKCB,SLC2A1,SLC2A4,BACE1,MAPK14,HRAS,PLAT,PLG,MAPK3,RELA,RPS6KB1,PDE10A
GO:0044853	plasma membrane raft	-16.97457999	ADRA1B,ADRA1A,CAV1,CHRNA7,CTNNB1,HAS2,HTR2A,INSR,JAK2,KCNMA1,NOS3,PRKACA,MAPK1,MAPK3,SCN5A,SELE,SLC2A1,SLC6A3,SRC,TGFBR2,HDAC6,LRRK2

Table S4. The information of the top 10 GO-MF pathways obtained through screening.

Term	Description	LogP	Symbols
GO:0004672	protein kinase activity	-61.50879764	ABL1,GRK2,AKT1,ALK,ARAF,AXL,CCND1,BRAF,BTK,CAMK2B,CCND3,CDK1,CDK2,CDK4,CDK5,CDK6,CHEK1,CHUK,MAP3K8,MAPK14,CSNK2A1,DAPK1,DYRK1A,EGFR,EPHB4,ERBB2,ERBB3,ERN1,FLT1,FLT3,FLT4,MTOR,GRK6,GSK3B,IGF1R,IKBKB,INSR,JAK1,JAK2,JAK3,KDR,LCK,LIMK1,MET,MYLK,NEK1,NEK2,PDGFRA,PDGFRB,PDPK1,PIK3CA,PIK3CB,PIM1,PIK3CG,PLK1,PRKACA,PRKCA,PRKCB,PRKCD,PRKCE,PKN1,PRKDC,MAPK1,MAPK3,MAPK8,MAPK9,MAPK10,MAP2K1,MAP2K2,EIF2AK2,PTK2,RAF1,RET,ROCK1,RPS6KA1,RPS6KA3,RPS6KB1,CCL2,MAP2K4,SRC,SYK,TGFBR2,TOP1,WEE1,CDC7,CDK5R1,MAPKAPK2,CHEK2,LRRK2,PIM3,ADK,HK2,PFKFB3,PIK3CD,PIK3R1,CCNE1
GO:0016773	phosphotransferase activity, alcohol group as acceptor	-60.12592552	ABL1,ADK,GRK2,AKT1,ALK,ARAF,AXL,CCND1,BRAF,BTK,CAMK2B,CCND3,CDK1,CDK2,CDK4,CDK5,CDK6,CHEK1,CHUK,MAP3K8,MAPK14,CSNK2A1,DAPK1,DYRK1A,EGFR,EPHB4,ERBB2,ERBB3,ERN1,FLT1,FLT3,FLT4,MTOR,GRK6,GSK3B,HK2,IGF1R,IKBKB,INSR,JAK1,JAK2,JAK3,KDR,LCK,LIMK1,MET,MYLK,NEK1,NEK2,PDGFRA,PDGFRB,PDPK1,PFKFB3,PIK3CA,PIK3CB,PIM1,PIK3CD,PIK3CG,PIK3R1,PLK1,PRKACA,PRKCA,PRKCB,PRKCD,PRKCE,PKN1,PRKDC,MAPK1,MAPK3,MAPK8,MAPK9,MAPK10,MAP2K1,MAP2K2,EIF2AK2,PTK2,RAF1,RET,ROCK1,RPS6KA1,RPS6KA3,RPS6KB1,CCL2,MAP2K4,SRC,SYK,TGFBR2,TOP1,WEE1,CDC7,CDK5R1,MAPKAPK2,CHEK2,LRRK2,PIM3
GO:0016301	kinase activity	-58.37869695	ABL1,ADK,GRK2,AKT1,ALK,ARAF,AXL,CCND1,BRAF,BTK,CAMK2B,CCND3,CCNE1,CDK1,CDK2,CDK4,CDK5,CDK6,CHEK1,CHUK,MAP3K8,MAPK14,CSNK2A1,DAPK1,DYRK1A,EGFR,EPHB4,ERBB2,ERBB3,ERN1,FLT1,FLT3,FLT4,MTOR,GRK6,GSK3B,HK2,IGF1R,IKBKB,INSR,JAK1,JAK2,JAK3,KDR,LCK,LIMK1,MET,MYLK,NEK1,NEK2,PDGFRA,PDGFRB,PDPK1,PFKFB3,PIK3CA,PIK3CB,PIM1,PIK3CD,PIK3CG,PIK3R1,PLK1,PRKACA,PRKCA,PRKCB,PRKCD,PRKCE,PKN1,PRKDC,MAPK1,MAPK3,MAPK8,MAPK9,MAPK10,MAP2K1,MAP2K2,EIF2AK2,PTK2,RAF1,RET,ROCK1,RPS6KA1,RPS6KA3,RPS6KB1,CCL2,MAP2K4,SRC,SYK,TGFBR2,TOP1,WEE1,CDC7,CDK5R1,MAPKAPK2,CHEK2,LRRK2,PIM3

GO:0019900	kinase binding	-43.04734157	ABL1,ACTA2,PARP1,ADRA2A,AKT1,APP,BAD,CCND1,BCL2L1,CASP1,CASP9,CAV1,CCNA2,CCNB1,CCND2,CCND3,CCNE1,CDC25A,CDC25B,CDKN1A,MAPK14,CTNNB1,E2F1,EGFR,ERBB2,ESR1,FNTA,GRB2,NR3C1,GSK3B,GSTP1,HIF1A,HSF1,HSPB1,HSP90AA1,HSP90AB1,IKBKB,ITGB3,JAK2,KIF11,LCK,LDHB,MAPT,NPM1,PCNA,PDGFRB,PIK3R1,PLG,PLK1,PPARA,PRKACA,PRKCB,PRKCD,PKN1,MAPK8,MAP2K1,PTK2,PTPN1,PTPN2,PTPN6,RB1,RELA,RPS6KA3,SCN5A,SLC2A1,STAT3,SYK,TNNI3,TOP2A,TP53,CDK5R1,MAPKAPK2,HDAC9,HDAC5,TRAP1,CHEK2,SIRT1,BACE1,PTPN22,NOX4,HDAC7,UGT1A10,UGT1A7
GO:0004674	protein serine/threonine kinase activity	-40.4211162	ABL1,GRK2,AKT1,ARAF,BRAF,CAMK2B,CCND3,CDK1,CDK2,CDK4,CDK5,CDK6,CHEK1,CHUK,MAP3K8,MAPK14,CSNK2A1,DAPK1,DYRK1A,EGFR,ERN1,MTOR,GRK6,GSK3B,IKBKB,LIMK1,MYLK,NEK1,NEK2,PDPK1,PIK3CA,PIM1,PIK3CG,PLK1,PRKACA,PRKCA,PRKCB,PRKCD,PRKCE,PKN1,PRKDC,MAPK1,MAPK3,MAPK8,MAPK9,MAPK10,MAP2K1,MAP2K2,EIF2AK2,RAF1,ROCK1,RPS6KA1,RPS6KA3,RPS6KB1,MAP2K4,SYK,TGFBR2,TOP1,CDC7,MAPKAPK2,CHEK2,LRRK2,PIM3
GO:0019901	protein kinase binding	-39.89278831	ABL1,ACTA2,PARP1,ADRA2A,AKT1,APP,BAD,CCND1,BCL2L1,CASP9,CAV1,CCNA2,CCNB1,CCND2,CCND3,CCNE1,CDC25A,CDC25B,CDKN1A,MAPK14,CTNNB1,E2F1,ERBB2,ESR1,FNTA,GRB2,NR3C1,GSK3B,GSTP1,HIF1A,HSF1,HSPB1,HSP90AA1,HSP90AB1,IKBKB,ITGB3,JAK2,KIF11,LCK,MAPT,NPM1,PCNA,PDGFRB,PIK3R1,PLK1,PPARA,PRKACA,PRKCB,PRKCD,PKN1,MAPK8,MAP2K1,PTK2,PTPN1,PTPN2,PTPN6,RELA,RPS6KA3,SCN5A,STAT3,SYK,TNNI3,TOP2A,TP53,CDK5R1,MAPKAPK2,HDAC9,HDAC5,TRAP1,CHEK2,SIRT1,BACE1,NOX4,HDAC7,UGT1A10,UGT1A7
GO:0106310	protein serine kinase activity	-35.22223946	AKT1,ARAF,BRAF,CAMK2B,CDK1,CDK2,CDK4,CDK5,CDK6,CHEK1,MAP3K8,MAPK14,CSNK2A1,DAPK1,DYRK1A,ERN1,MTOR,GSK3B,IKBKB,LIMK1,NEK1,NEK2,PDPK1,PIK3CA,PIK3CB,PIM1,PIK3CG,PLK1,PRKACA,PRKCA,PRKCB,PRKCD,PRKCE,PKN1,PRKDC,MAPK1,MAPK3,MAPK8,MAPK9,MAPK10,MAP2K1,MAP2K2,EIF2AK2,RAF1,ROCK1,RPS6KA1,RPS6KA3,RPS6KB1,MAP2K4,CDC7,MAPKAPK2,CHEK2,LRRK2,PIM3
GO:0008134	transcription factor binding	-29.96267607	PARP1,AHR,AR,BCL2,RUNX2,RUNX1T1,MAPK14,CTNNB1,E2F1,ELK1,ESR1,ESRRB,FLT3,FOS,MTOR,NR3C1,GSK3B,HDAC1,HDAC2,HIF1A,HSF1,HSPB1,JUN,NR3C2,MYC,NFE2L2,NFKB1,NFKBIA,NPM1,PCNA,PGR,PIM1,PPARA,PPARD,PPARG,PRKCB,PKN1,PRKDC,MAPK3,PTPN2,RB1,RELA,RORA,RXRA,STAT1,STAT3,TERT,THRB,TP53,NR1H2,NCOA1,NR1I2,HDAC9,HDAC4,HDAC6,HDAC5,NCOA2,KDM1A,SIRT1,HDAC7,HDAC8,APEX1,APP,CDK1,EGFR,MPO,RXRB,TOP1,TOP2A,TOP2B,PARP2,NR1H3

GO:0016491	oxidoreductase activity	-28.97062739	ADH1C,ALDH2,AKR1B1,ALOX12,ALOX5,ALOX15,APEX1,CBR1,CYP1A1,CYP1A2,CYP1B1,CYP2C19,CYP3A4,CYP17A1,CYP19A1,CYP27B1,CYP51A1,DAO,AKR1C1,DHCR7,DHCR24,DHODH,NQO1,FASN,G6PD,GSTM2,GSTP1,HMGCR,HMOX1,HSD3B1,HSD3B2,HSD11B1,HSD17B1,HSD17B3,IMPDH1,IMPDH2,LDHA,LDHB,MAOA,MAOB,MPO,COX2,NOS2,NOS3,POR,PTGS1,PTGS2,SOD1,SORD,SQLE,TBXAS1,TYR,VCAM1,XDH,AKR1C3,PTGES,AKR1A1,KDM1A,DUOX2,NOX4,AKR1B10,FTO,NCF1
GO:0004713	protein tyrosine kinase activity	-28.12400291	ABL1,ALK,AXL,BTK,DYRK1A,EGFR,EPHB4,ERBB2,FLT1,FLT3,FLT4,MTOR,IGF1R,INSR,JAK1,JAK2,JAK3,KDR,LCK,MET,NEK1,PDGFRA,PDGFRB,PRKCD,MAP2K1,MAP2K2,EIF2AK2,PTK2,RET,MAP2K4,SRC,SYK,WEE1,TGFBR2,COL1A1,COL3A1,ERBB3,IGFBP3,ITGB3,SCN5A

Table S5. The information of the top 25 KEGG pathways obtained through screening.

Term	Description	LogP	Symbols
hsa04151	PI3K-Akt signaling pathway	-60.09818458	AKT1|BAD|CCND1|BCL2|BCL2L1|CASP9|CCND2|CCND3|CCNE1|CDK2|CDK4|CDK6|CDKN1A|CHRM1|CHRM2|CHUK|COL1A1|EGF|EGFR|ERBB2|ERBB3|FLT1|FLT3|FLT4|MTOR|GRB2|GSK3B|HRAS|HSP90AA1|HSP90AB1|IGF1R|IGF2|IKBKB|IL2|IL4|IL6|INSR|ITGB3|JAK1|JAK2|JAK3|KDR|MCL1|MDM2|MET|MYC|NFKB1|NOS3|PDGFRA|PDGFRB|PDPK1|PGF|PIK3CA|PIK3CB|PIK3CD|PIK3CG|PIK3R1|PRKCA|PKN1|MAPK1|MAPK3|MAP2K1|MAP2K2|PTEN|PTK2|RAF1|RELA|RET|RPS6KB1|RXRA|SPP1|SYK|TP53|HSP90B1|VEGFA
hsa04010	MAPK signaling pathway	-45.68850638	AKT1|ARAF|BRAF|CACNA1S|CASP3|CDC25B|CHUK|MAP3K8|MAPK14|EGF|EGFR|ELK1|ERBB2|ERBB3|FLT1|FLT3|FLT4|FOS|GRB2|HRAS|HSPB1|IGF1R|IGF2|IKBKB|IL1A|IL1B|INSR|JUN|KDR|MAPT|MET|MYC|NFKB1|PDGFRA|PDGFRB|PGF|PRKACA|PRKCA|PRKCB|MAPK1|MAPK3|MAPK8|MAPK9|MAPK10|MAP2K1|MAP2K2|RAF1|RASA1|RELA|RET|RPS6KA1|RPS6KA3|MAP2K4|TGFB1|TGFBR2|TNF|TP53|VEGFA|MAPKAPK2
hsa04012	ErbB signaling pathway	-41.2340924	ABL1|AKT1|ARAF|BAD|BRAF|CAMK2B|CDKN1A|EGF|EGFR|ELK1|ERBB2|ERBB3|MTOR|GRB2|GSK3B|HRAS|JUN|MYC|PIK3CA|PIK3CB|PIK3CD|PIK3R1|PRKCA|PRKCB|MAPK1|MAPK3|MAPK8|MAPK9|MAPK10|MAP2K1|MAP2K2|PTK2|RAF1|RPS6KB1|MAP2K4|SRC
hsa04066	HIF-1 signaling pathway	-41.22561339	AKT1|BCL2|CAMK2B|CDKN1A|EDN1|EGF|EGFR|ERBB2|FLT1|MTOR|HIF1A|HK2|HMOX1|IFNG|IGF1R|IL6|INSR|LDHA|LDHB|NFKB1|NOS2|NOS3|SERPINE1|PFKFB3|PIK3CA|PIK3CB|PIK3CD|PIK3R1|PRKCA|PRKCB|MAPK1|MAPK3|MAP2K1|MAP2K2|RELA|RPS6KB1|SLC2A1|STAT3|VEGFA
hsa04068	FoxO signaling pathway	-40.43035046	AKT1|ARAF|CCND1|BRAF|CCNB1|CCND2|CDK2|CDKN1A|CHUK|MAPK14|EGF|EGFR|GRB2|HRAS|IGF1R|IKBKB|IL6|IL10|INSR|MDM2|PDPK1|PIK3CA|PIK3CB|PIK3CD|PIK3R1|PLK1|MAPK1|MAPK3|MAPK8|MAPK9|MAPK10|MAP2K1|MAP2K2|PTEN|RAF1|SLC2A4|STAT3|TGFB1|TGFBR2|CCNB2|SIRT1
hsa04926	Relaxin signaling pathway	-39.41124682	ACTA2|ADCY2|AKT1|COL1A1|COL3A1|MAPK14|EDN1|EGFR|FOS|GRB2|HRAS|JUN|MMP1|MMP2|MMP9|MMP13|NFKB1|NFKBIA|NOS2|NOS3|PIK3CA|PIK3CB|PIK3CD|PIK3R1|PRKACA|PRKCA|MAPK1|MAPK3|MAPK8|MAPK9|MAPK10|MAP2K1|MAP2K2|RAF1|RELA|MAP2K4|SRC|TGFB1|TGFBR2|VEGFA

hsa04919	Thyroid hormone signaling pathway	-37.68248136	AKT1|BAD|CCND1|CASP9|CTNNB1|ESR1|MTOR|GSK3B|HDAC1|HDAC2|HIF1A|HRAS|ITGB3|MDM2|MYC|PDPK1|PIK3CA|PIK3CB|PIK3CD|PIK3R1|PRKACA|PRKCA|PRKCB|MAPK1|MAPK3|MAP2K1|MAP2K2|RAF1|RXRA|RXRB|RXRG|SLC2A1|SRC|STAT1|THRB|TP53|NCOA1|NCOA2
hsa04660	T cell receptor signaling pathway	-34.75218952	AKT1|CD40LG|CDK4|CHUK|MAP3K8|MAPK14|CSF2|FOS|GRB2|GSK3B|HRAS|IFNG|IKBKB|IL2|IL4|IL10|JUN|LCK|NFKB1|NFKBIA|PDPK1|PIK3CA|PIK3CB|PIK3CD|PIK3R1|MAPK1|MAPK3|MAPK8|MAPK9|MAPK10|MAP2K1|MAP2K2|PTPN6|RAF1|RELA|TNF
hsa04917	Prolactin signaling pathway	-34.57009095	AKT1|CCND1|CCND2|MAPK14|CYP17A1|ESR1|ESR2|FOS|GRB2|GSK3B|HRAS|IRF1|JAK2|NFKB1|PIK3CA|PIK3CB|PIK3CD|PIK3R1|MAPK1|MAPK3|MAPK8|MAPK9|MAPK10|MAP2K1|MAP2K2|RAF1|RELA|SRC|STAT1|STAT3
hsa04014	Ras signaling pathway	-32.82113805	ABL1|AKT1|BAD|BCL2L1|CHUK|EGF|EGFR|ELK1|FLT1|FLT3|FLT4|GRB2|HRAS|HTR7|IGF1R|IGF2|IKBKB|INSR|KDR|MET|NFKB1|PDGFRA|PDGFRB|PGF|PIK3CA|PIK3CB|PIK3CD|PIK3R1|PLA2G1B|PRKACA|PRKCA|PRKCB|MAPK1|MAPK3|MAPK8|MAPK9|MAPK10|MAP2K1|MAP2K2|RAF1|RASA1|RELA|VEGFA|RASSF1
hsa04722	Neurotrophin signaling pathway	-32.16587841	ABL1|AKT1|BAD|BAX|BCL2|BRAF|CAMK2B|MAPK14|GRB2|GSK3B|HRAS|IKBKB|JUN|NFKB1|NFKBIA|PDPK1|PIK3CA|PIK3CB|PIK3CD|PIK3R1|PRKCD|MAPK1|MAPK3|MAPK8|MAPK9|MAPK10|MAP2K1|MAP2K2|RAF1|RELA|RPS6KA1|RPS6KA3|TP53|MAPKAPK2
hsa04062	Chemokine signaling pathway	-31.91216401	ADCY2|GRK2|AKT1|BAD|BRAF|CHUK|GRK6|GRB2|CXCL2|GSK3B|HRAS|IKBKB|CXCL8|CXCR1|CXCL10|JAK2|JAK3|NFKB1|NFKBIA|PIK3CA|PIK3CB|PIK3CD|PIK3CG|PIK3R1|PRKACA|PRKCB|PRKCD|MAPK1|MAPK3|MAP2K1|PTK2|RAF1|RELA|ROCK1|CCL2|CXCL11|SRC|STAT1|STAT3|NCF1
hsa04915	Estrogen signaling pathway	-28.45238854	ADCY2|AKT1|BCL2|CTSD|EGFR|ESR1|ESR2|FOS|GRB2|HRAS|HSP90AA1|HSP90AB1|JUN|MMP2|MMP9|NOS3|OPRM1|PGR|PIK3CA|PIK3CB|PIK3CD|PIK3R1|PRKACA|PRKCD|MAPK1|MAPK3|MAP2K1|MAP2K2|RAF1|SRC|HSP90B1|NCOA1|NCOA2
hsa04630	JAK-STAT signaling pathway	-28.04522766	AKT1|CCND1|BCL2|BCL2L1|CCND2|CCND3|CDKN1A|CSF2|EGF|EGFR|MTOR|GRB2|HRAS|IFNG|IL2|IL4|IL6|IL10|JAK1|JAK2|JAK3|MCL1|MYC|PDGFRA|PDGFRB|PIK3CA|PIK3CB|PIM1|PIK3CD|PIK3R1|PTPN2|PTPN6|RAF1|STAT1|STAT3
hsa04910	Insulin signaling pathway	-27.25179973	ACACA|AKT1|ARAF|BAD|BRAF|ELK1|FASN|MTOR|GRB2|GSK3B|HK2|HRAS|IKBKB|INSR|PDPK1|PIK3CA|PIK3CB|PIK3CD|PIK3R1|PRKACA|MAPK1|MAPK3|MAPK8|MAPK9|MAPK10|MAP2K1|MAP2K2|PTPN1|PYGL|RAF1|RPS6KB1|SLC2A4
hsa04370	VEGF signaling pathway	-27.03966218	AKT1|BAD|CASP9|MAPK14|HRAS|HSPB1|KDR|NOS3|PIK3CA|PIK3CB|PIK3CD|PIK3R1|PRKCA|PRKCB|MAPK1|MAPK3|MAP2K1|MAP2K2|PTGS2|PTK2|RAF1|SRC|VEGFA|MAPKAPK2

hsa04664	Fc epsilon RI signaling pathway	-26.84784507	AKT1|ALOX5|BTK|MAPK14|CSF2|GRB2|HRAS|IL4|PDPK1|PIK3CA|PIK3CB|PIK3CD|PIK3R1|PRKCA|MAPK1|MAPK3|MAPK8|MAPK9|MAPK10|MAP2K1|MAP2K2|RAF1|MAP2K4|SYK|TNF
hsa04015	Rap1 signaling pathway	-24.42295665	ADCY2|ADORA2A|ADORA2B|AKT1|BRAF|CNR1|MAPK14|CTNNB1|DRD2|EGF|EGFR|FLT1|FLT4|HRAS|IGF1R|INSR|ITGB3|KDR|MET|PDGFRA|PDGFRB|PGF|PIK3CA|PIK3CB|PIK3CD|PIK3R1|PRKCA|PRKCB|MAPK1|MAPK3|MAP2K1|MAP2K2|RAF1|SRC|VEGFA
hsa04662	B cell receptor signaling pathway	-22.01814018	AKT1|BTK|CHUK|FOS|GRB2|GSK3B|HRAS|IKBKB|JUN|NFKB1|NFKBIA|PIK3CA|PIK3CB|PIK3CD|PIK3R1|PRKCB|MAPK1|MAPK3|MAP2K1|MAP2K2|PTPN6|RAF1|RELA|SYK
hsa04072	Phospholipase D signaling pathway	-21.22799505	ADCY2|AKT1|AVPR2|EGF|EGFR|F2|MTOR|GRB2|GRM4|HRAS|CXCL8|CXCR1|INSR|PDGFRA|PDGFRB|PIK3CA|PIK3CB|PIK3CD|PIK3CG|PIK3R1|PRKCA|MAPK1|MAPK3|MAP2K1|MAP2K2|RAF1|SYK|AGPAT2
hsa04150	mTOR signaling pathway	-19.35667685	AKT1|BRAF|CHUK|MTOR|GRB2|GSK3B|HRAS|IGF1R|IKBKB|INSR|PDPK1|PIK3CA|PIK3CB|PIK3CD|PIK3R1|PRKCA|PRKCB|MAPK1|MAPK3|MAP2K1|MAP2K2|PTEN|RAF1|RPS6KA1|RPS6KA3|RPS6KB1|TNF
hsa04550	Signaling pathways regulating pluripotency of stem cells	-14.81288061	AKT1|MAPK14|CTNNB1|ESRRB|GRB2|GSK3B|HRAS|IGF1R|JAK1|JAK2|JAK3|MYC|PIK3CA|PIK3CB|PIK3CD|PIK3R1|MAPK1|MAPK3|MAP2K1|MAP2K2|RAF1|STAT3
hsa04933	AGE-RAGE signaling pathway in diabetic complications	-60.14856582	AKT1|BAX|CCND1|BCL2|CASP3|CDK4|COL1A1|COL3A1|MAPK14|EDN1|F3|HRAS|ICAM1|IL1A|IL1B|IL6|CXCL8|JAK2|JUN|MMP2|NFKB1|NOS3|SERPINE1|PIK3CA|PIK3CB|PIM1|PIK3CD|PIK3R1|PRKCA|PRKCB|PRKCD|PRKCE|MAPK1|MAPK3|MAPK8|MAPK9|MAPK10|RELA|CCL2|SELE|STAT1|STAT3|TGFB1|TGFBR2|THBD|TNF|VCAM1|VEGFA|NOX4
hsa04668	TNF signaling pathway	-44.28672671	AKT1|BIRC2|CASP3|CASP7|CASP8|CHUK|MAP3K8|MAPK14|CSF2|EDN1|FOS|CXCL2|ICAM1|IKBKB|IL1B|IL6|CXCL10|IRF1|JUN|MMP3|MMP9|MMP14|NFKB1|NFKBIA|PIK3CA|PIK3CB|PIK3CD|PIK3R1|MAPK1|MAPK3|MAPK8|MAPK9|MAPK10|MAP2K1|PTGS2|RELA|CCL2|SELE|MAP2K4|ADAM17|TNF|VCAM1
hsa04657	IL-17 signaling pathway	-36.140213	CASP3|CASP8|CHUK|MAPK14|CSF2|FOS|CXCL2|GSK3B|HSP90AA1|HSP90AB1|IFNG|IKBKB|IL1B|IL4|IL6|CXCL8|CXCL10|JUN|MMP1|MMP3|MMP9|MMP13|NFKB1|NFKBIA|MAPK1|MAPK3|MAPK8|MAPK9|MAPK10|PTGS2|RELA|CCL2|TNF|HSP90B1
